# Effects of the Sex Chromosome Complement, XX, XO, or XY, on the Transcriptome and Development of Mouse Oocytes During Follicular Growth

**DOI:** 10.3389/fgene.2021.792604

**Published:** 2021-12-20

**Authors:** Wataru Yamazaki, Dunarel Badescu, Seang Lin Tan, Jiannis Ragoussis, Teruko Taketo

**Affiliations:** ^1^ Department of Surgery, McGill University, Montreal, QC, Canada; ^2^ Research Institute of McGill University Health Centre, Montreal, QC, Canada; ^3^ Department of Human Genetics, McGill University, Montreal, QC, Canada; ^4^ McGill University Genome Centre, Montreal, QC, Canada; ^5^ Department of Obstetrics and Gynecology, McGill University, Montreal, QC, Canada; ^6^ OriginElle Fertility Clinic and Women’s Health Centre, Montreal, QC, Canada; ^7^ Department of Biology, McGill University, Montreal, QC, Canada

**Keywords:** ovary, oocyte, XO female, XY sex reversal, transcriptome, mitochondria, BMP15, KDM5B

## Abstract

The sex chromosome complement, XX or XY, determines sexual differentiation of the gonadal primordium into a testis or an ovary, which in turn directs differentiation of the germ cells into sperm and oocytes, respectively, in eutherian mammals. When the X monosomy or XY sex reversal occurs, XO and XY females exhibit subfertility and infertility in the mouse on the C57BL/6J genetic background, suggesting that functional germ cell differentiation requires the proper sex chromosome complement. Using these mouse models, we asked how the sex chromosome complement affects gene transcription in the oocytes during follicular growth. An oocyte accumulates cytoplasmic components such as mRNAs and proteins during follicular growth to support subsequent meiotic progression, fertilization, and early embryonic development without *de novo* transcription. However, how gene transcription is regulated during oocyte growth is not well understood. Our results revealed that XY oocytes became abnormal in chromatin configuration, mitochondria distribution, and *de novo* transcription compared to XX or XO oocytes near the end of growth phase. Therefore, we compared transcriptomes by RNA-sequencing among the XX, XO, and XY oocytes of 50–60 µm in diameter, which were still morphologically comparable. The results showed that the X chromosome dosage limited the X-linked and autosomal gene transcript levels in XO oocytes whereas many genes were transcribed from the Y chromosome and made the transcriptome in XY oocytes closer to that in XX oocytes. We then compared the transcript levels of 3 X-linked, 3 Y-linked and 2 autosomal genes in the XX, XO, and XY oocytes during the entire growth phase as well as at the end of growth phase using quantitative RT-PCR. The results indicated that the transcript levels of most genes increased with oocyte growth while largely maintaining the X chromosome dosage dependence. Near the end of growth phase, however, transcript levels of some X-linked genes did not increase in XY oocytes as much as XX or XO oocytes, rendering their levels much lower than those in XX oocytes. Thus, XY oocytes established a distinct transcriptome at the end of growth phase, which may be associated with abnormal chromatin configuration and mitochondria distribution.

## Introduction

The sex chromosome complement, XX or XY, determines sexual differentiation of the gonadal primordium into a testis or an ovary, which in turn directs differentiation of the germ cells into spermatogenesis or oogenesis, respectively, in eutherian mammals (McLaren, 1988; Berta et al., 1990; Koopman et al., 1990). When gonadal sex is reversed, however, the germ cell sex becomes discordant with the chromosomal sex. Both sex-reversed XX males and XY females encounter infertility, indicating that functional germ cell differentiation requires the presence of proper sex chromosome complement. While essential roles of the Y chromosome in spermatogenesis have been well defined, the cause of infertility in the XY oocyte has remained an enigma (Taketo, 2015; Yamauchi et al., 2016).

In fetal ovaries, the germ cells enter meiosis to become oocytes and go through the Meiotic Prophase I (MPI), in which homologous chromosomes pair and recombine. In the XX germ cells, one of the two X-chromosomes is initially inactivated like in somatic cells, but it becomes reactivated prior to the entry into meiosis, and the two transcriptionally active X-chromosomes efficiently pair and recombine during the MPI progression (de Napoles et al., 2007; Sugimoto and Abe, 2007; Chuva De Sousa Lopes et al., 2008). In perinatal ovaries, the oocytes reach the end of MPI and form primordial follicles with the neighboring granulosa cells. The oocytes remain arrested at this stage in the ovarian reserve in the entire reproductive life. Upon puberty, a cohort of primordial follicles is recruited into the growth phase, during which granulosa cells vigorously proliferate to form multiple layers while the oocyte increases in volume and undergoes active transcription and translation. At the end of growth phase, the oocytes, now named fully-grown (FG) or germinal vesicle (GV) oocytes, shut down transcription and become competent for going through the first meiotic division and reaching the second meiotic metaphase (MII). Upon fertilization, the MII-oocytes undergo the second meiotic division to initiate embryonic development. The oocytes of XY female mice can go through oogenesis to reach the MII-stage, but fail in embryonic development, resulting in poor fertility or infertility (reviewed by (Taketo, 2015)). We predict that one dosage of X chromosome and the presence of Y chromosome together alter the mRNAs and proteins accumulated during follicular growth and make the XY oocyte incompetent for embryonic development. The oocytes of XO female mice are competent for embryonic development. However, the normal XX oocyte carries two transcriptionally active X chromosomes, and if and how the absence of one X chromosome affects the transcriptome in the XO oocyte remains to be clarified.

In the present study, we asked how the sex chromosome complement affects the oocyte during follicular growth. We used the B6.Y^TIR^ congenic mouse strain to produce XY females. The B6.Y^TIR^ mouse was established by placing the Y chromosome from a variant of *Mus musculus domesticus* caught in Tirano, Italy (TIR) onto the C57BL/6J (B6) genetic background by repeating backcross (Eicher et al., 1982; Nagamine et al., 1987). The gonadal sex is reversed because the SRY protein encoded on the Y^TIR^ chromosome has polymorphic differences from that encoded on the Y^B6^ chromosome, and fails to sufficiently upregulate its target *Sox9* gene on B6, which is essential for testicular differentiation (Coward et al., 1994; Taketo et al., 2005; Park et al., 2011). All stages of follicles can be seen in prepubertal B6.Y^TIR^ (XY herein) ovaries, but very few MII-oocytes complete the second meiotic division or initiate embryonic development after fertilization (Taketo-Hosotani et al., 1989; Amleh et al., 1996; Villemure et al., 2007; Zhu et al., 2017). The failure in embryonic development can be attributed to cytoplasmic defects; when the XY oocyte nucleus has been transferred into an enucleated XX oocyte, the reconstructed oocyte develops into a healthy pup after *in vitro* fertilization and embryo transfer (Obata et al., 2008). This rescue is more efficient when the oocyte nucleus is replaced at the GV stage than at the MII-stage, suggesting that the XY ooplasm becomes defective largely by the end of growth phase. By 2 months of age, the XY female mouse retains very few or no follicles and fails to initiate estrous cycle (Taketo-Hosotani et al., 1989; Amleh and Taketo, 1998).

In humans, monosomy 45.X (XO) embryos rarely survive *in utero*, and those who have reached the term suffer from congenital abnormalities and infertility, termed Turner’s syndrome (Turner, 1938; Singh and Carr, 1966; Ogata and Matsuo, 1995; Hook and Warburton, 2014). In mice, by contrast, most XO embryos survive to term and show no gross anomalies except for lower body weights than their XX littermates (Cattanach, 1962; Burgoyne et al., 1983). These striking somatic differences between the two species can be attributed to the greater number of genes that escape from X chromosome inactivation in humans compared to mice (Berletch et al., 2010; Yang et al., 2010; Tukiainen et al., 2017). Moreover, XO female mice are fertile, suggesting that one X chromosome is sufficient for rendering the oocyte to become competent for embryonic development in the mouse. Nonetheless, XX and XO oocytes are not equal when exogenous genes are expressed (Vernet et al., 2014b; Hamada et al., 2020). In the current study, we produced XO females by using the male mouse carrying *Patchy Fur* (*Paf*) mutation on the X chromosome, which was provided on the C3H/HeSnJ background from the Jackson Laboratory. The *Paf* mutation in males causes a high incidence of X-Y non-disjunction at the first meiotic division and sires XO daughters (Lane and Davisson, 1990; Korobova et al., 1998; Burgoyne and Evans, 2000). In order to compare XO oocytes with XY oocytes on the same genetic background, we backcrossed the *Paf* mutation onto B6 (Vaz et al., 2020). The XO female in this breeding scheme inherits the maternal wild-type X chromosome, like the B6.Y^TIR^ female, and exhibits an early oocyte loss, infertility or subfertility with high embryonic lethality, the features of which are shared with Turner’s syndrome in humans (Vaz et al., 2020).

In the current study, we first compared transcriptomes in XX, XO, and XY oocytes of the largest size in which no difference was yet apparent at morphological or global transcriptional activity. We then monitored the changes in transcript levels of selected genes during the entire growth phase. The results show dominant effects of the X chromosome dosage in XO oocytes and a unique transcriptome landscape associated with Y-linked gene transcription in XY oocytes.

## Materials and Methods

### Mice

All animal experiments were performed in accordance with the Canadian Council on Animal Care and approved by the McGill University Animal Care Committee. B6.Y^TIR^ males (N65-76 backcross generations) were crossed with B6 females (Jackson Laboratory, Bar Harbor, ME) to produce XY females and their XX littermates. *Paf* breeding pair on the C3H/HeSnJ background was purchased from the Jackson Laboratory and backcrossed to B6 in our mouse colony. *Paf* carrier males (N6-8 backcross generations) were crossed with B6 females to produce XO females. The day of delivery was defined as 0 days postpartum (dpp). For the use of pups at 18 dpp or younger, their ear punches and tail biopsies were taken for identifying XY and XO females, respectively, 1 day prior to the experiment. For the use of pups at 25 dpp or older, ear punches and tail biopsies were taken upon weaning at 20–25 dpp. Ear punches were digested in a fresh lysis buffer containing 25 mM NaOH and 0.2 mM EDTA disodium (pH 8.0) at 95°C for 40 min, and subject to PCR amplification of the Y-linked *Zfy* gene using the conditions described previously. From tail biopsies, total RNAs were extracted using TRIzol (Invitrogen, Burlington, ON) according to the manufacture’s protocol, and dissolved in RNase-free water. The RNA samples were subject to cDNA synthesis using M-MLV Reverse Transcriptase (Invitrogen, Thermo-Fisher Scientific, St Laurent, QC) according to the manufacture’s protocol and subsequently to PCR amplification of the *Xist* transcript (Kay et al., 1994), which was present in XX females and absent in XO females.

### Collection of Oocytes During the Growth Phase

Ovaries were collected from XX, XO, and XY females at 8, 10, 12, and 18 dpp, and individually dissociated into single cells by the method previously described (Taketo, 2012) with minor modifications. In brief, ovaries were treated with 0.1% collagenase (Sigma-Aldrich, St. Louis, MO) in Eagle’s minimum essential medium containing Hank’s salts supplemented with 0.25 mM Hepes buffer (both from GIBCO, Long Island, NY) (named MEM-H) for 30 min at 37°C, followed by 0.25% trypsin (Worthington Biochemical, Lakewood, NJ) in Rinaldini solution for 20 min at 37°C. After washings, ovaries were dissociated in phosphate buffer saline (PBS) in microfuge tubes by repeated pipetting, and then centrifuged. The pellet was resuspended in 0.5–2.0 ml (depending on genotypes and age of females) M2 medium, transferred into a Petri dish, and the diameters of oocytes were measured under an inverted microscope (Leica DM IRB, Germany).

### Collection of Fully-Grown oocytes

XX, XO, and XY females at 25–29 dpp were injected intraperitoneally with 10 IU equine chronic gonadotropin (Sigma-Aldrich) and euthanized 46 h later. FG (GV-stage) oocytes surrounded with cumulus cells (COC) and spontaneously denuded oocytes (DNO) were collected after puncturing large antral follicles with a pair of 26-gauge needles in the M2 medium supplemented 5.0 μM milrinone (Sigma-Aldrich). The oocytes in COCs were stripped off the cumulus cells by repeating pipetting through a fine glass needle.

### Nuclear Transfer in Fully-Grown Oocytes

The oocytes denuded from COCs were incubated in MEM-α supplemented with 5% FBS, 75 μg/ml penicillin G potassium salt and 50 μg/ml streptomycin (all from GIBCO) and 5.0 μM milrinone for 1 h at 37°C with 5% CO_2_ in a humidified atmosphere to create the perivitelline space between the zona pellucida and oocyte. The zona pellucida over the perivitelline space was slit with a fine glass knife in M2 containing milrinone under an inverted microscope. The nucleus was removed with a small amount of ooplasm and transferred into another enucleated oocyte using a fine glass pipette using CellTram^®^ vario (Eppendorf, Hamburg, Germany). The recipient oocytes were placed between two gold electrodes, 0.5 mm apart, in a fusion chamber filled with M2 containing milrinone, and electro-pulsed at 55 V for 50 μs in ECFG21 Super Electro Cell Fusion Generator (NEPAGENE, Chiba, Japan). Oocytes were then incubated for fusion in MEM-α supplemented with FBS, antibiotics and milrinone for 1 h. The reconstructed oocytes were cultured in MEM-α supplemented with 250 μM sodium pyruvate (GIBCO), 5% FBS, penicillin and streptomycin, 300 ng/ml FSH (Sigma-Aldrich) and 5.0 μM milrinone for 13–14 h.

### Chromatin Configuration

Oocytes collected from COCs, spontaneously denuded oocytes, and oocytes in the growth phase (50–60 µm in diameter) were fixed with 2% paraformaldehyde (Electron Microscopy Sciences, Hatfield, PA) in microtubule stabilizing buffer (Messinger and Albertini, 1991) at room temperature for 1 h. The oocytes were then washed three times in PBS containing 3% BSA and blocked in PBS containing 5% FBS and 0.1% Triton X-100 overnight at 4°C. After three washes, oocytes were mounted in Prolong Antifade Mounting Medium containing DAPI (Molecular Probe, Eugene, OR) on Plus-coated histology slides (Thermo-Fisher Scientific). Images were captured and examined under a confocal microscope (Zeiss LSM 780, Germany).

### Mitochondria Distribution

FG-oocytes and oocytes in the growth phase (40–50 µm in diameter) were stained with 200 and 400 nM, respectively, Mito-Tracker Orange CMTMRos (Molecular Probes, Themo-Fisher Sientific) at 37°C for 30 min with 5% CO_2_ in a humidified atmosphere, followed by fixation, washing and blocking as described above. After three washes, the oocytes were mounted in Prolong Antifade Mounting Medium containing DAPI on Plus-coated histology slides. Images were captured and examined under a confocal microscope. Fluorescence intensity of Mito-Tracker Orange CMTMRos was measured by ZEN software (Carl ZEISS MicroImaging).

### *De Novo* Transcription

The *de novo* transcriptional activity in FG-oocytes and oocytes in the growth phase (50–70 μm in diameter) was detected using Click-iT™ RNA Alexa Fluor™ 488 Imaging kit (Invitrogen, Thermo-Fisher Scientific). In brief, oocytes were incubated in 1 mM 5-ethynyl uridine (EU) for 1 h, followed by fixation, washing and blocking as described above. After three washes, the oocytes were fluorescence stained according to the manufacture’s protocols and mounted in Prolong Antifade Mounting Medium containing DAPI on Plus-coated histology slides. Images were captured and analysed under a confocal microscope. EU fluorescence intensity in the nucleus was measured in individual oocytes by ZEN software. For FG-oocytes, the mean intensity of the non-surrounded-nucleolus (NSN)-type XX oocytes was set as 1.0 and the relative intensity in each oocyte was calculated in every experiment. For the oocytes in the growth phase, the mean intensity of the XX oocytes of 60–65 μm in diameter was set as 1.0 for calculating the relative intensity in all oocytes in every experiment.

### Immunofluorescence Staining of H3K4me3 in Oocytes

FG-oocytes and oocytes in the growth phase (50–70 μm in diameter) were fixed as described above and incubated with rabbit monoclonal anti tri-methyl-histone H3 (Lys4) antibody (#9751, Cell Signaling Technology, New England Biolabs, Whitby, ON) (1:200) at 4°C overnight. After three washes, oocytes were incubated with goat-anti-rabbit IgG-FITC (Jackson ImmunoResearch, West Grove, PA) (1:500) at room temperature for 1 h. After three washes, the oocytes were mounted in Prolong Antifade Mounting Medium containing DAPI on Plus-coated histology slides. Images were captured and examined under a confocal microscope. Fluorescence intensity was measured in individual oocytes by ZEN software. For the oocytes in the growth phase, the mean intensity of the XX oocytes of 60–70 μm in diameter was set as 1.0 for calculating the relative intensity in all oocytes in every experiment. For FG-oocytes, the mean fluorescence intensity in the surrounded-nucleolus (SN)-type XX oocytes was set as 1.0.

### RNA Preparation and Sequencing

In order to perform transcriptomic analysis of small numbers of oocytes, we applied the Smart-seq3 method (Hagemann-Jensen et al., 2020) with some modifications as follows. A total of 30 oocytes (10 oocytes of 50–55 μm and 20 oocytes of 55–60 µm in diameter) in 5 µL volume per sample were pooled into 10 µL of lysis buffer [4.54 µM polyT primer, 0.128% Triton-X 100, 3 mM each dNTP, 1 U/μl RNase inhibitor, 4.7 attomoles (diluted by 220,000 fold) of “ERCC spike-in Mix 1” (ThermoFisher #4456740)] in biological triplicate for each genotype. The diluted stock of “ERCC spike-in Mix 1” should correspond to 3–6% of total reads sequenced per sample of 30 oocytes based on theoretical calculations. Lysis was carried out in a thermocycler at 72°C for 3 min, 4°C for 10 min, and 25°C for 1 min, and the lysed samples were stored at −80°C. cDNA synthesis and library preparation was performed with an in-house Smart-seq3 protocol using modifications for first and second strand cDNA synthesis as follows: 1) oligo-dTVN: /5Me-isodC/AGATGTGTATAAGAGACAGN(12)ACT(30)VN: 2) Template Switching Oligo (TSO): /5Me-isodC//iisodG//iMe-isodC/AGATGTGTATAAGAGACAGN(12)ACGCrGrGrG and Invitrogen SuperScript IV Reverse Transcriptase (Thermo-Fisher Scientific): 3) The Nextera PCR primer TCG​TCG​GCA​GCG​TCA​GAT​GTG​TAT​AAG​AGA​CAG was used for single primer cDNA amplification, using Advantage 2 Polymerase Mix (Takara). NGS libraries were generated as described (Hagemann-Jensen et al., 2020) and quality control was performed using electropherogram profiling on a Caliper HS DNA. Chip Sequencing was performed on an Illumina NovaSeq 6000 SP lane in Paired-end 150 bp mode.

### Data Processing and Bioinformatic Analysis

The primary reads were aligned to the GRCm38 mouse genome and transcriptome from Gencode, using Hisat2, in stranded and paired-end mode (Leek and Storey, 2007; Zhang et al., 2019). Reads from the 5′ exons were demultiplexed and UMIs counted, using regular expressions according to the 5′ end sequencing patterns on an Apache Spark cluster (Zaharia et al., 2016). The read counts, corresponding to 5′ UMIs, were normalized using ERCC spike-ins (Ritchie et al., 2015). Quality control was performed using principal component analysis and heatmap analysis of the top 500 highly and differentially expressed genes, using a distance matrix of the Spearman correlation coefficient. Batch effects were assessed using Surrogate Variable Analysis (*sva*, R bioconductor package) (Leek and Storey, 2007), and included as covariates into the linear model using the R Bioconductor *limma* package for differential expression (Ritchie et al., 2015). We used the *lv2Transformer*, an improved version of the default log-voom transformation, for *limma* provided by the *countTransformers* R package (Zhang et al., 2019). For all other purposes including clustering, batch effects were regressed-out using *cleaningY* function of the *jaffelab* R package (Collado-Torres et al. https://github.com/LieberInstitute/jaffelab, R package version 0.99.31).

All expressed genes present in the normalized expression matrix were used for scatterplots in three-way comparisons among XX, XO, and XY oocytes. Differentially expressed genes (DEGs) were selected in three-way comparisons at *p* < 0.05 and their overlapping was analysed in Venn diagrams as follows. Directional over or under expressed DEGs were obtained from the three-way comparisons, totaling 6 DEG lists. According to the focus on gain or loss by the X chromosome or the Y chromosome, we selected 4 comparison groups out of the 6 DEG lists. A schematic diagram is presented with arrows pointing towards the directional over expression in each figure. Venn diagrams were constructed by intersection of 4 DEG lists.

For Y-linked gene analyses, *p*-values were adjusted to Rate False Discover Rate (FDR) using Benjamini-Hochberg (https://www.statisticshowto.com/benjamini-hochberg-procedure/).

For the X-linked DEGs identified as X chromosome dosage dependent, the ratio of transcript levels in XO and XY oocytes against that in XX oocytes was calculated using a lognormal distribution, fitted using R-package *EnvStats*, giving the mean, standard deviation and confidence internals. The ratio of all X-linked vs. autosomal gene transcript levels was calculated using bootstrap analysis framework from *boot* R-package as follows. Gene expression was separated into 6 equally sized bins, and genes were assigned to each category. Sampling with replacement, with a size of 100, repeated for 5,000 times, was performed from the X-linked genes and autosomal genes separately. The median for each extracted sample was calculated, and then a ratio of the X:A transcript levels was calculated. Its distribution was represented as boxplot, for each expression level bin and genotype. A similar procedure was applied for calculating the ratio of mean transcript levels in XO and XY oocytes against that in XX oocytes, using only the highly expressed genes in bin 6. Comparison of X, Y and autosomal homologous gene transcript levels was also performed using the R statistical framework, based on the geometric mean of the replicate data (Olivier et al., 2008), having lognormal standard deviation intervals represented.

### qRT-PCR

Total RNA was extracted from 10–30 pooled oocytes of each genotype and size range with RNeasy RNA isolation kit (Qiagen, Montreal, QC), and subject to cDNA synthesis using oligo(dT) (Life Technologies, Thermo-Fisher Scientific) in total 20 µL. One µl of each cDNA solution was subject to qPCR of a gene in duplicate using a FastStart Essential DNA Green Master with LightCycler^®^ 96 Instrument (Roche, Mannheim, Germany). Primers used for qPCR are listed in Supplementary Table S1. Endogenous *Ppia* levels were used for normalization in each cDNA sample and qRT-PCR procedure.

### Statistical Analyses

All experiments except for RNA-Seq were repeated at least three times. When proportions of oocytes in different categories were compared between two genotypes, χ^2^-test was used. Where values were normally distributed, data of three genotypes were presented as means ± SEM and statistically analyzed by two-sided *t*-test or one-way ANOVA followed by Tukey’s honestly significant difference (HSD) test. Where values did not fit into normal distribution, data were presented in a 25–75% percent box plot with the median in line, and statistical difference was evaluated by the Dunn’s multiple comparison test.

## Results

### Chromatin Configuration

**FIGURE 1:**
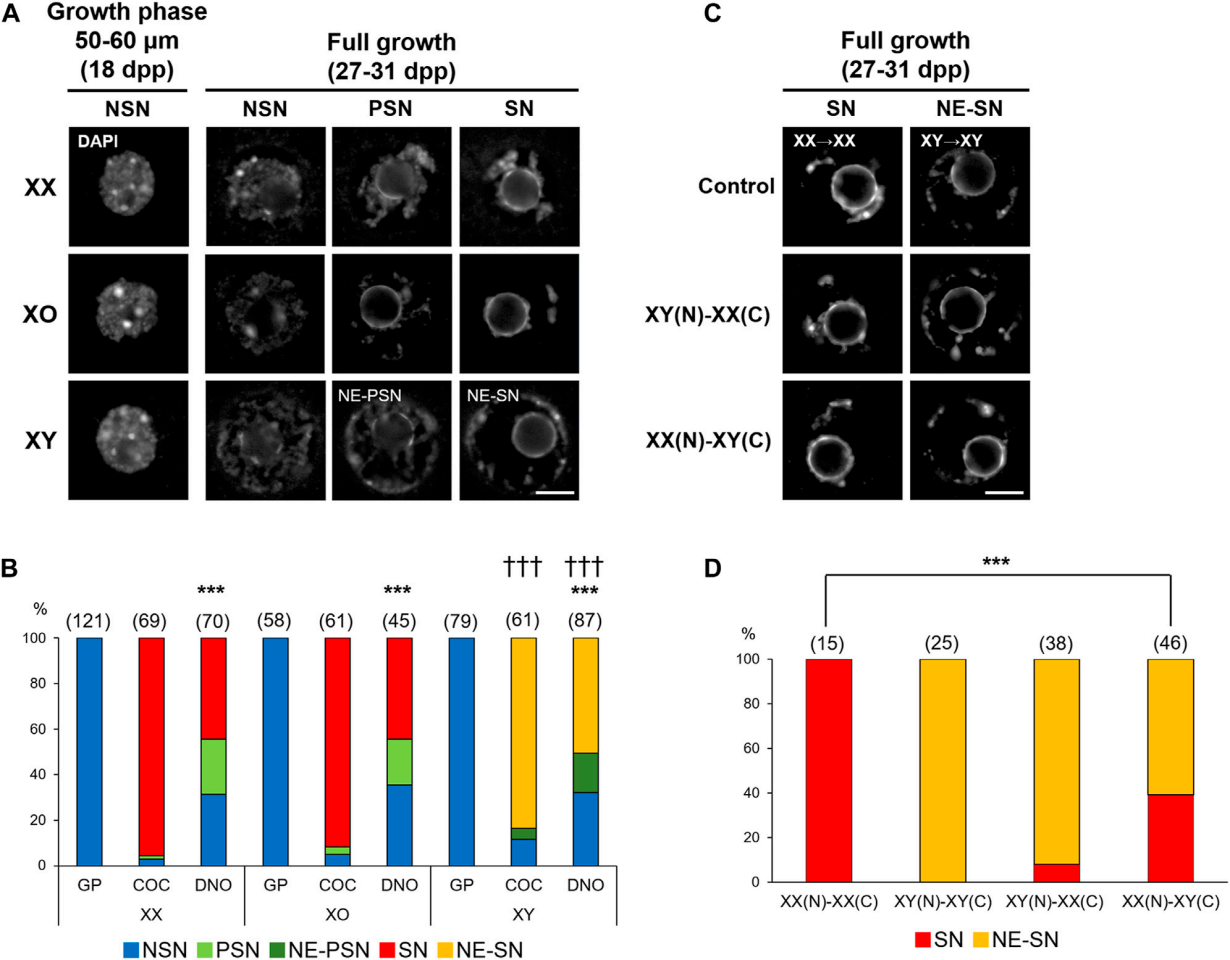
Chromatin configuration in XX, XO, and XY oocytes in the growth phase. **(A)** Representative images of chromatin configuration with DAPI staining. XX and XO oocytes showed three types; non-surrounded nucleolus (NSN), partially surrounded nucleolus (PSN), and surrounded nucleolus (SN), whereas XY oocytes showed chromatin condensation along the nuclear envelop (NE) in addition to PSN or SN-type condensation. Scale bar, 10 μm. **(B)** Percentages of oocytes with different types of chromatin configuration. Oocytes examined were either in the growth phase (GP), at full growth collected from cumulus-oocyte complexes (COC), or spontaneously denuded at the collection of COCs (DNO). The total number of oocytes examined is given above each column. *** and ††† indicate statistical differences compared with the oocytes from COCs of the same genotype and with XX and XO oocytes in the same category, respectively, at *p* < 0.001 by *X* ^2^-test. **(C)** Representative images of chromatin configuration in the nucleus (N) after transfer into an enucleated oocyte cytoplasm (C). Scale bar, 10 μm. **(D)** Percentages of oocytes with different types of chromatin configuration in the reconstructed oocytes. The total number of oocytes examined is given above each column. *** indicates statistical difference from the control oocytes, XX(N)-XX(C), at *p* < 0.001 by X^2^-test.

We first compared the chromatin configuration in XX, XO, and XY oocytes with DAPI staining. As previously reported (Debey et al., 1993; Zuccotti et al., 1995), we observed decondensed chromatin configuration in the nuclei of most XX oocytes in the mid growth phase (50–60 µm), named non-surrounded nucleolus (NSN), and progressive condensation of chromatin around the nucleolus, named partially surrounded nucleolus (PSN) to surrounded nucleolus (SN), by the end of growth phase (Figure 1A). When the XX FG-oocytes collected from COCs were examined, 95.7% showed SN-type chromatin configuration while the rest showed PSN- and NSN-type chromatin configuration (Figure 1B). However, when the spontaneously denuded FG-oocytes (DNO) were examined, only 44.3% were of SN-type while 24.3 and 31.4% were of PSN- and NSN-type, respectively. The frequencies of oocytes with the three types of chromatin configuration were significantly different between the oocytes of the two origins at *p* < 0.001 by χ^2^-test. Similarly, 91.8% of the XO FG-oocytes collected from COCs were of SN-type while only 44.4% of the spontaneously denuded XO FG-oocytes were SN-type with a significant difference at *p* < 0.001. By contrast, although most XY oocytes in the growth phase were seen with NSN-type chromatin configuration, comparable with XX and XO oocytes, none of the XY FG-oocytes showed typical PSN- or SN-type chromatin configuration. Instead, 83.6 and 4.9% of the XY oocytes collected from COCs showed chromatin condensation along the nuclear envelop (NE) in addition to SN- and PSN-type condensation, respectively. NE-type chromatin condensation was never seen in XX or XO oocytes. The remaining 11.5% of the XY oocytes from COCs showed NSN-type chromatin configuration. Spontaneously denuded XY FG-oocytes also showed NE-SN- and NE-PSN-type chromatin configuration in addition NSN-type chromatin configuration, but neither typical SN- nor PSN-type. These results suggest that the chromatin condensation was not affected by the lack of one X chromosome in XO oocytes while it became abnormal in XY oocytes at the end of growth phase.

Chromatin configuration can be determined within the nucleus or affected by cytoplasmic components. To distinguish these two possibilities, we performed nuclear transfer between XX and XY FG-oocytes collected from COCs. In controls, the nucleus of an XX oocyte was transferred into an enucleated XX oocyte, referred as XX(N)-XX(C), and the nucleus of an XY oocyte was transferred into an enucleated XY oocyte, as XY(N)-XY(C). All control reconstructed oocytes showed SN- and NE-SN-type chromatin configuration as expected for XX and XY oocytes without manipulation (Figures 1C,D). When the nucleus of an XY oocyte was transferred into an enucleated XX oocyte, presented as XY(N)-XX(C), 92.1% of the reconstructed oocytes showed NE-SN-type nuclei and were not statistically different from control XY(N)-XY(C) oocytes. However, when the nucleus of an XX oocyte was transferred into an enucleated XY oocyte, referred as XX(N)-XY(C), 60.9% of the reconstructed oocytes showed NE-SN-type chromatin configuration with a significant difference from control XX(N)-XX(C) oocytes at *p* < 0.001. These results suggest that the cytoplasm of XY oocytes was mainly responsible for the abnormal chromatin condensation along the nuclear envelop. However, the chromatin configuration in the XY nucleus appears to have been irreversibly altered by the end of growth phase and could not be corrected by brief contact with the XX cytoplasm. Therefore, we cannot exclude the possibility that the XY nucleus per se also contributed to the abnormal chromatin configuration.

### Distribution of Mitochondria With Active Membrane Potential

**FIGURE 2:**
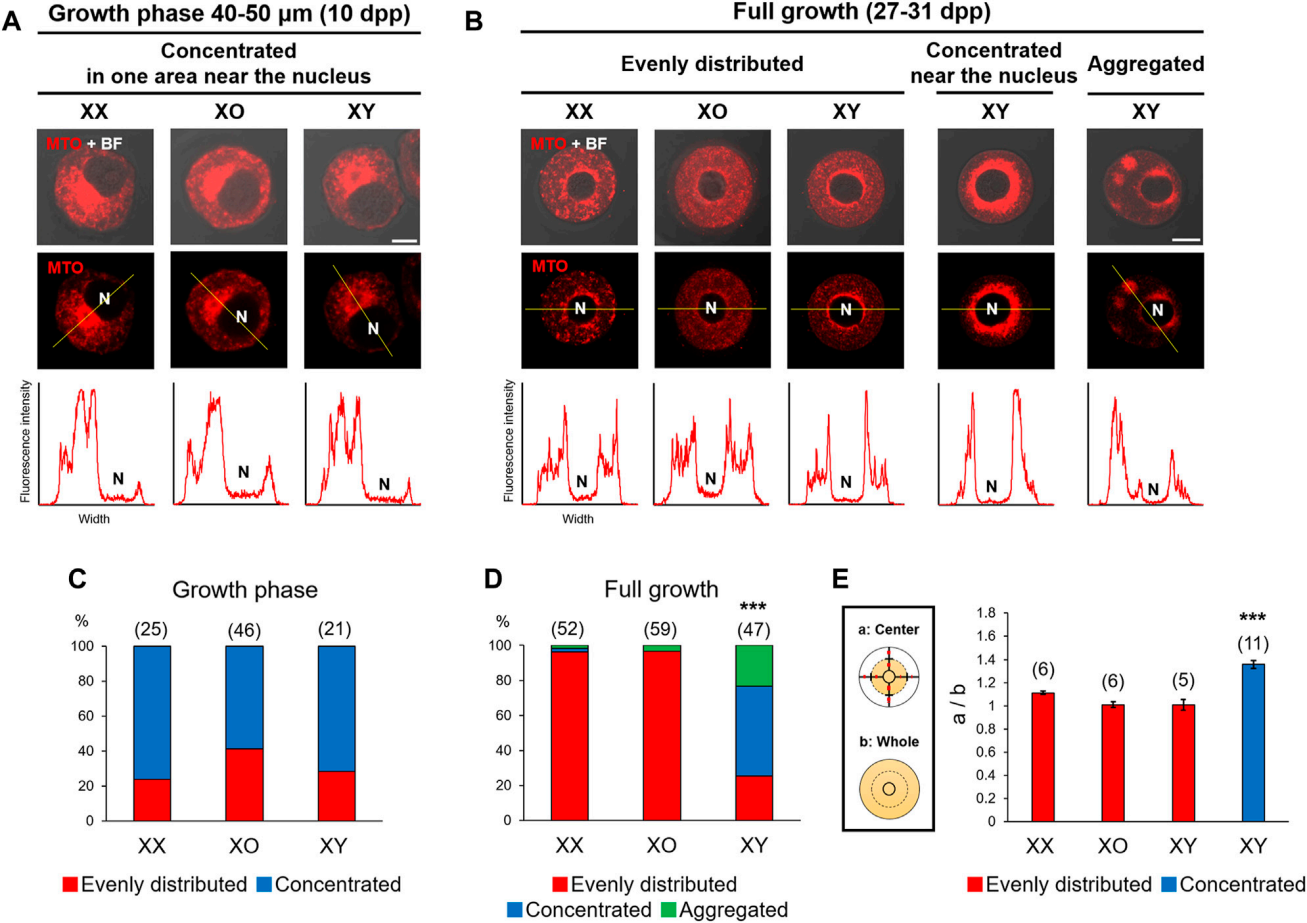
Distribution of mitochondria with high membrane potential in XX, XO, and XY oocytes in the growth phase. **(A,B)** Representative images of mitochondria staining with MitoTracker Orange (MTO). The image taken with phase contrast in the bright field (BF) is overlayed on the top panel. The intensity of fluorescence signals along the yellow line drawn in the picture is shown beneath each picture. N, nucleus. Scale bar, 10 μm. **(A)** XX and XY oocytes of 40–50 µm. **(B)** XX, XO, and XY FG-oocytes. In the majority of XX and XO oocytes, MTO staining was evenly distributed in the cytoplasm except for the narrow area around the nucleus. In XY oocytes, MTO staining was more concentrated near the nucleus or aggregated in the cytoplasm. Scale bar, 20 μm. **(C)** Percentages of XX and XY oocytes in the growth phase with different types of mitochondria distribution. The total number of oocytes examined is given above each column. **(D)** Percentages of XX, XO, and XY FG-oocytes with different types of mitochondria distribution. *** indicates statistical difference from XX or XO oocytes at *p* < 0.001 by X^2^-test. **(E)** Ratios of the total intensity of fluorescence signals in the central cytoplasmic area to that in the whole area. Each oocyte was divided by the half distance between the nuclear envelop and the ooplasmic membrane. Each column indicates the mean ± SEM. The number of oocytes examined is given above each column. *** indicates statistical difference in the XY oocyte with concentrated mitochondria, compared to XX, XO, and XY oocytes with evenly distributed mitochondria at *p* < 0.001 by one-way ANOVA followed by Tukey’s honestly significant difference (HSD) test.

Chromatin configuration is known to be affected by cytoplasmic components (Inoue et al., 2008) such as mitochondria, which can be easily visualized. Accordingly, we examined the distribution of mitochondria by staining with MitoTracker Orange, which indicates the high mitochondrial membrane potential. As shown in Figures 2A,C, metabolically active mitochondria were concentrated in an area near the nucleus of 60–80% oocytes of 40–50 μm, regardless of the genotype. When the nucleus was positioned off the center, mitochondria were concentrated in the wider side of cytoplasm. With further oocyte growth, mitochondria became evenly distributed in the cytoplasm of most XX and XO oocytes while the nucleus relocated to the centre (Figures 2B,D). By contrast, only 25.5% of XY FG-oocytes showed evenly distributed mitochondria while 51.1% showed highly concentrated mitochondria around the nucleus. The relative intensity of MTO fluorescence in the perinuclear area of these XY oocytes was significantly higher than in the oocytes which were categorized as “evenly distributed” (Figure 2E). The remaining 23.4% of XY oocytes showed mitochondria in a few large aggregates in the cytoplasm (Figures 2B,D). We also stained some oocytes with MitoTracker Green, which was independent of membrane potential, and found similar mitochondria distribution (not shown). Thus, mitochondria distribution was comparable in XX, XO, and XY oocytes during the growth phase and became abnormal in the XY oocytes near the end of growth phase.

### De Novo Transcription

**FIGURE 3:**
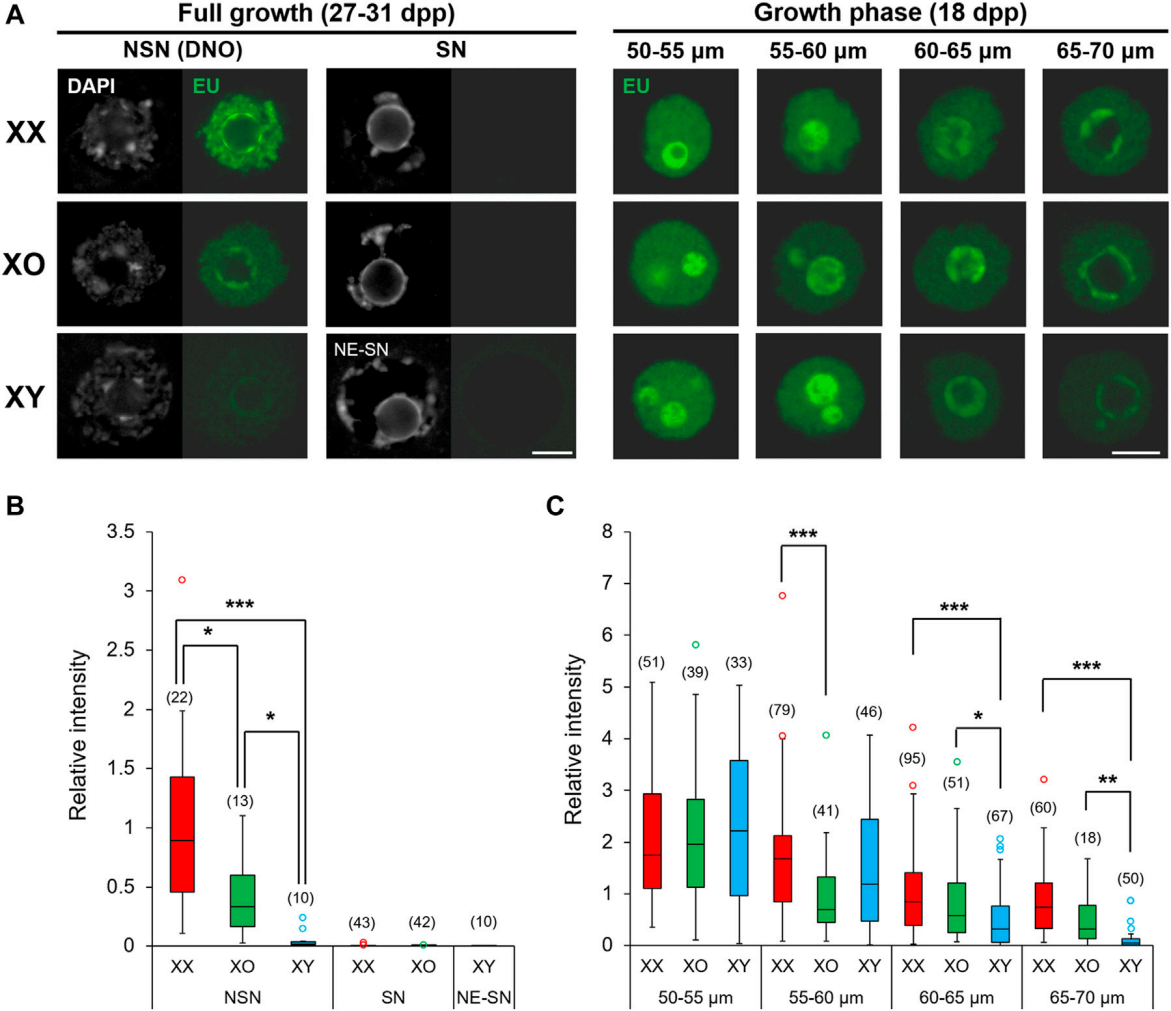
*De novo* transcription in XX, XO, and XY oocytes in the growth phase. **(A)** Representative images of chromatin configuration (DAPI) with NSN, SN or NE-SN-type and EU incorporation (EU) in XX, XO, and XY FG-oocytes. Scale bar, 10 μm. **(B,C)** The relative fluorescence intensity of EU incorporation in the fully grown oocytes with NSN, SN or NE-SN-type chromatin configuration **(B)** or during growth **(C)**. Each box plot indicates the median with 1st and 3rd quartiles. The thin vertical line indicates the range from minimum to maximum values. ° indicates outlier. The total number of oocytes examined is given above each column. *, **, and *** indicate statistical differences at *p* < 0.05, 0.01 and 0.001, respectively, by Dunn’s multiple comparison test.

The chromatin configuration, NSN or SN, in XX FG-oocytes is associated with transcriptional silencing and competence for embryonic development (Bouniol-Baly et al., 1999; de la Fuente and Eppig, 2001). In our results, XY FG-oocytes showed abnormal chromatin condensation along the nuclear envelope (NE), distinct from typical NSN- or SN-type. Therefore, we examined *de novo* transcription in these oocytes by EU incorporation (Figure 3A). De novo transcription was quiescent in XX and XO FG-oocytes with SN-type chromatin configuration as well as in XY FG-oocytes with NE-SN-type chromatin configuration. By contrast, EU staining intensity was diverse among the oocytes of three genotypes with NSN-type chromatin configuration; very high in XX oocytes, very low in XY oocytes, and intermediate in XO oocytes. We found large variability of fluorescence signal intensity among the oocytes of the same genotype, which often did not fit into the normal distribution. Therefore, we presented the results by the median and interquartile range (IQR) with minimum and maximum values, and statistically analysed the data by the Dunn’s multiple comparison test (Figure 3B). The results indicated significant differences in all three-way comparisons.

We then examined the oocytes during the growth phase and measured the relative intensity of EU staining (See Methods). The results showed that EU staining intensity was comparable among XX, XO and XY oocytes of 50–55 μm, the smallest size examined (Figure 3C). The EU staining intensity then gradually declined in XX oocytes with further growth to low but still detectable levels at 65–70 µm in diameter. For comparison, EU staining intensity significantly declined in XO oocytes of 55–60 µm compared to XX or XY oocytes of the same size and remained lower than XX oocytes although without significant difference afterwards. The EU staining intensity in XY oocytes was initially comparable with that in XX oocytes up to 55–60 μm, but it declined rapidly to become significantly lower than XX or XO oocytes at 60–65 µm. Thus, *de novo* transcription declined early in XO oocytes, while it declined slightly later but further in XY oocytes approaching the end of growth phase, compared to XX oocytes. Thus, both the absence of the second X chromosome and the presence of the Y chromosome affected the global transcription during oocyte growth.

### Differentially Expressed Genes in the Oocytes by RNA-Sequencing

To elucidate the role of sex chromosome complement in establishing the transcriptome in the oocytes during the growth phase, we analyzed differentially expressed genes (DEGs) by RNA-Sequencing (RNA-Seq) among XX, XO, and XY oocytes of 50–60 μm, all of which were still transcriptionally active (See above). To reduce the bias, 10 oocytes of 50–55 μm and 20 oocytes of 55–60 µm were pooled in each sample. The sequencing data, as well as full length coverage tracks, raw 5′ UMI, and normalized expression matrices reported in this study have been deposited in the Gene Expression Omnibus website with accession code GSE184153.

**FIGURE 4:**
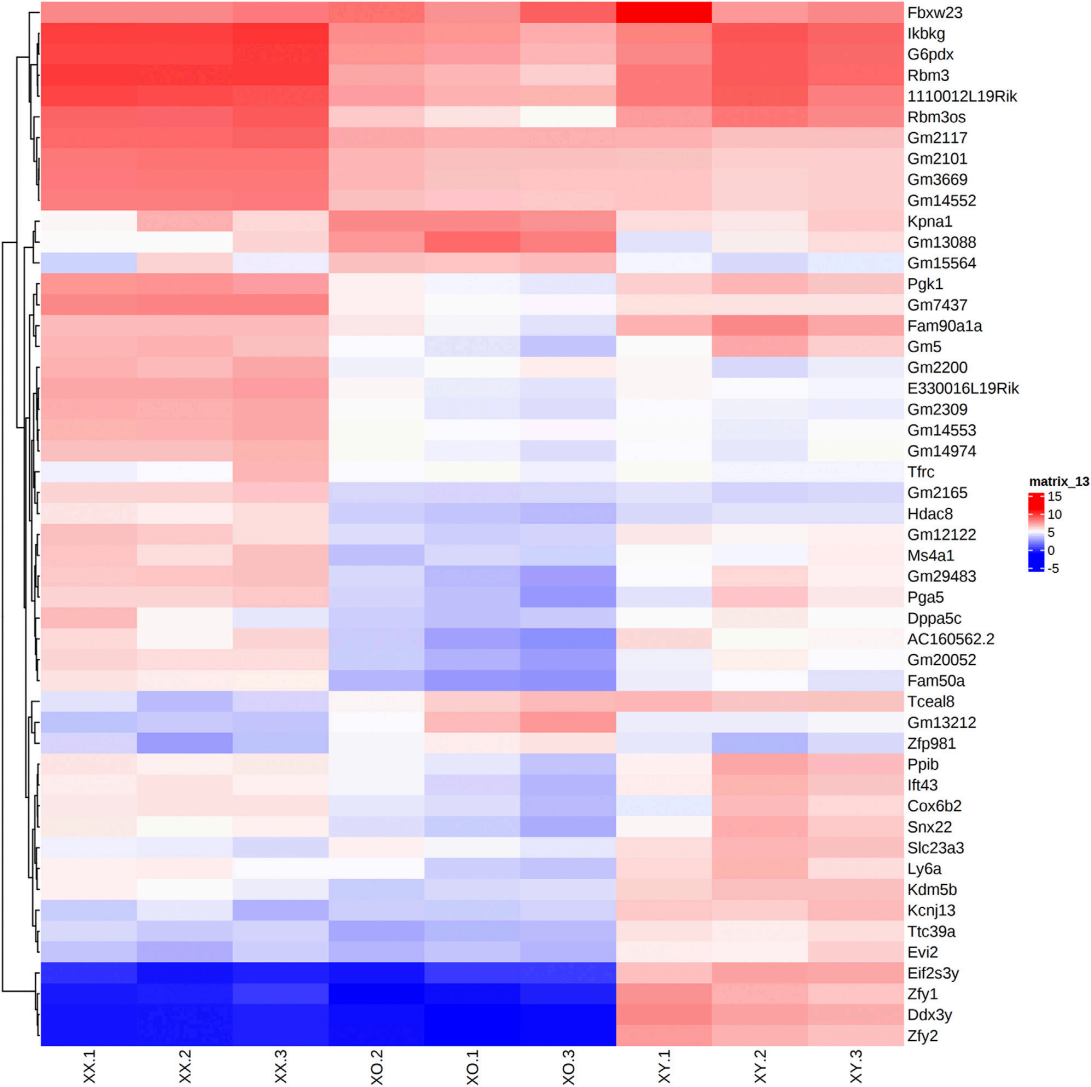
Heatmap (Log2CPM) of the top 50 highly and differentially expressed genes in biological triplicates of XX, XO, and XY oocytes, based on the distance matrix of the Spearman correlation coefficient.

**FIGURE 5:**
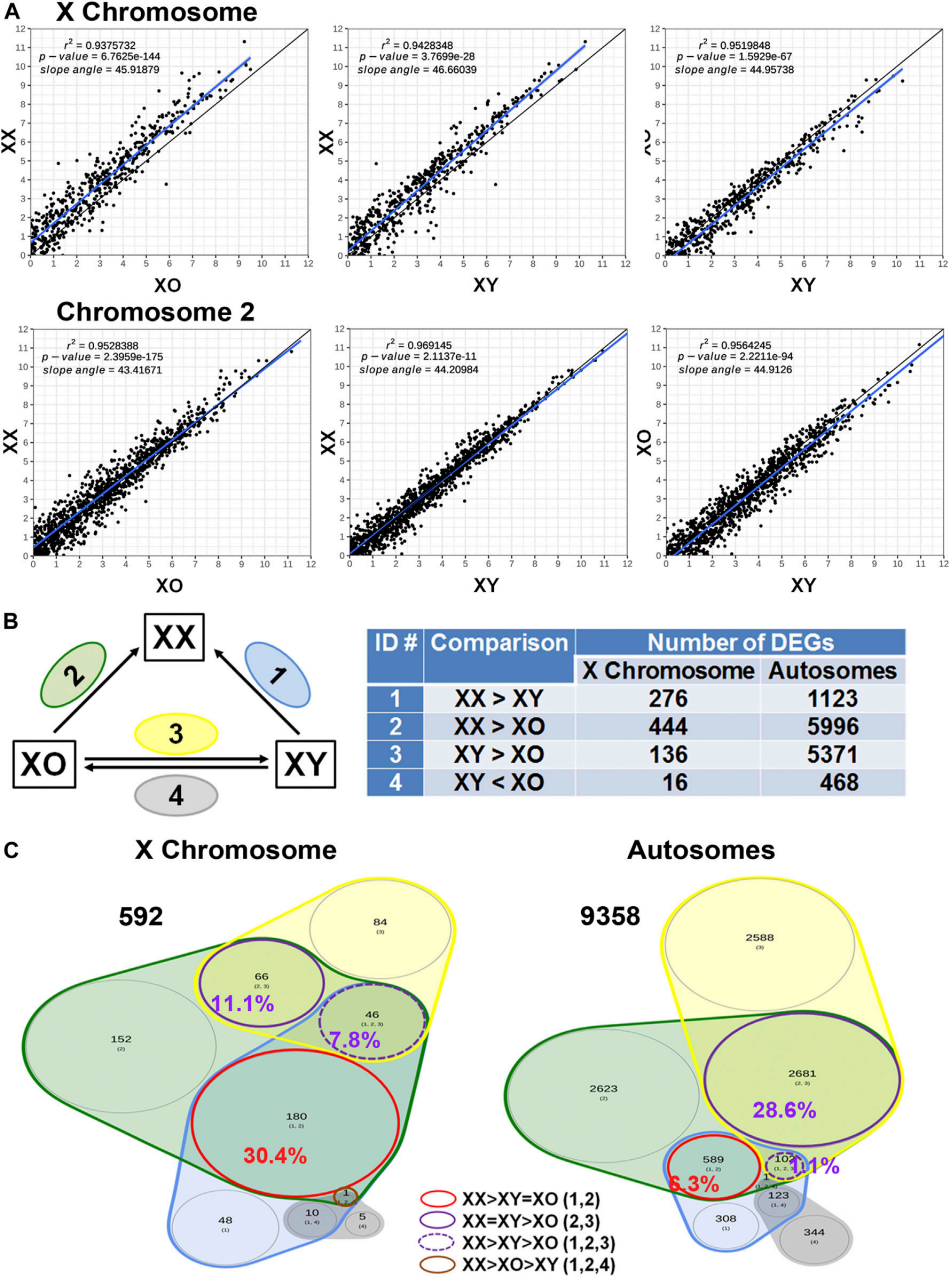
Sex chromosome dosage dependent differentially expressed genes (DEGs). **(A)** Scatterplots of all expressed genes on the X chromosome and Chromosome 2 in XX vs. XO, XX vs. XY, and XO vs. XY oocytes. **(B)** The total number of DEGs (*p* < 0.05) in four comparison groups with focus on gain by the second X chromosome. **(C)** Venn diagrams to indicate the overlapping of DEGs among four comparison groups. The area is proportional to the number of DEGs. Transcript levels of the genes in a red circle were higher in XX oocytes than those in XO or XY oocytes without difference between XO and XY oocytes. The genes in a solid purple circle were equally higher in XX and XY oocytes than XO oocytes. The genes in a broken purple circle were higher in the order of XX, XY, and XO oocytes. The single gene (*Bmp15*) in a brown circle was lower in XY oocytes than in XX or XO oocytes.

Across all three genotypes, a total of 28,051 genes were detected, while sample quality was interrogated using principal component analysis (not shown) and heatmap analysis of the 500 highly expressed and most variable genes, using a distance matrix of the Spearman correlation coefficient (Figure 4). The results showed the highest consistency among the biological replicates of the same genotype although XX1, XO2, and XY1 showed less similarity to their other two replicates. Transcript levels (5′UMI reads) of all X-linked genes were scatter-plotted in three-way comparisons, XX vs. XO, XX vs. XY, and XO vs. XY oocytes (Figure 5A), all of which showed strong coefficiency (*r* ^2^ > 0.937). We separated X-linked and autosomal genes because of the X chromosome dosage difference among the three genotypes. The vast majority of X-linked genes were in fact shifted upward from the 1:1 line in XX vs. XO and XX vs. XY oocytes whereas most genes were scattered around the 1:1 line in XO vs. XY oocytes. For comparison, most expressed genes from Chromosome 2 were scattered around the 1:1 line in all three-way comparisons. To reveal the X chromosome dosage effects, we selected DEGs at *p* < 0.05 in four-way comparison groups, 1) XX > XY, 2) XX > XO, 3) XY > XO, and 4) XY < XO, as given in Figure 5B. The total number of X-linked DEGs was 276, 444, 136, and 16, respectively, whereas the total number of autosomal DEGs was 1,123, 5,996, 5,371, and 468, respectively. Overlapping of these DEGs among four comparison groups was shown in Venn diagrams (Figure 5C), where the area is proportional to the number of DEGs.

### X Chromosome Dosage Dependent DEGs

We first asked how the X chromosome dosage reflected into X-linked gene transcript levels. Of the total 592 DEGs found in all four comparison groups, 180 (30.4%) DEGs had higher transcript levels in XX oocytes than in XO or XY oocytes without significant difference between XO and XY oocytes (red circle). These genes represent the DEGs which were predominant affected by the X chromosome dosage. Other genes may also be X chromosome dosage-dependent, but they were affected by other components such as the Y chromosome. For comparison, of the total 9,358 autosomal DEGs, only 589 (6.3%) showed the same relationship, confirming that transcript levels of the 180 X-linked genes represented the X chromosome dosage dependent DEGs. Examples are *Atrx*, which encodes a chromatin remodeling factor (de la Fuente et al., 2004b; Balboula et al., 2015), *Eif2s3x*, a translation initiation factor, and *Pdha1*, pyruvate dehydrogenase E1.

**FIGURE 6:**
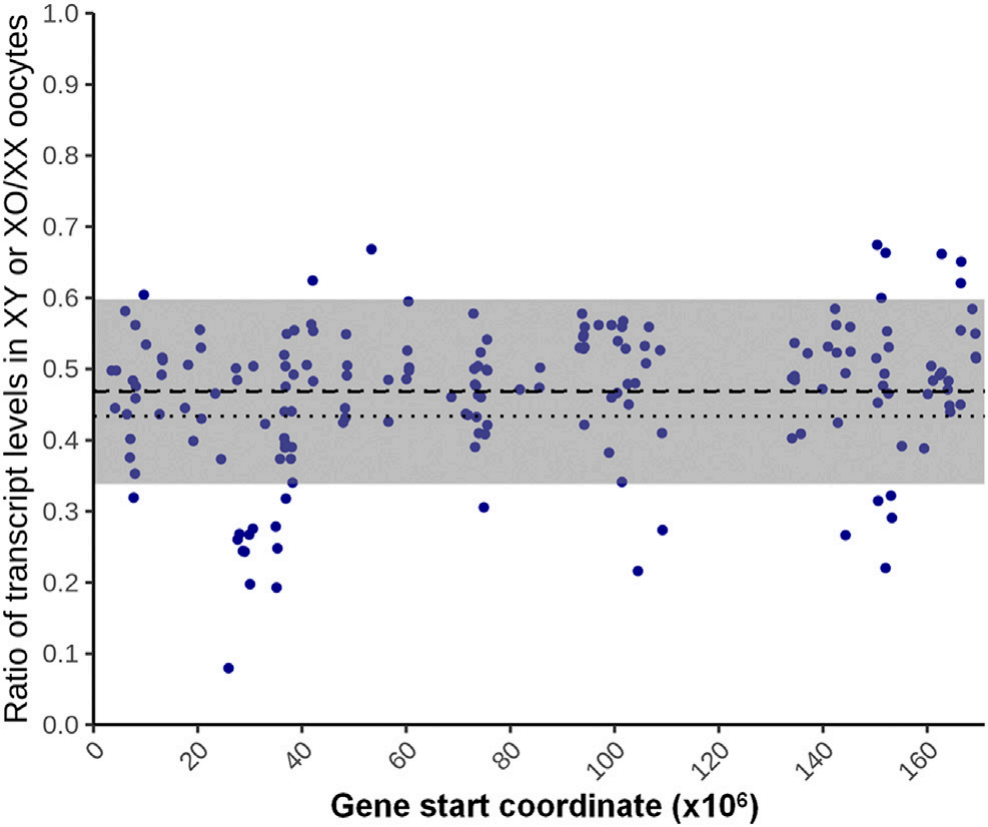
X chromosome dosage dependent differentially expressed genes (DEGs). The ratio of the average transcript levels of individual DEGs in XO and XY oocytes against those in XX oocytes. The X-axis indicates the distance from the proximal end of the X chromosome. The thick broken line indicates the geometrical mean, thin broken lines indicate the confidence of mean, and gray area indicates the mean ± SD.

For the 180 genes identified as X chromosome dosage dependent X-linked DEGs, we used a fitted lognormal distribution of the data, and calculated the ratio of geometric mean transcript levels in XO and XY oocytes against those in XX oocytes. We then plotted the ratios at their loci on the X chromosome, which clustered within 0.47 ± 0.13 (mean ± SD) (Figure 6). We also calculated the ratio of transcript levels in XO or XY oocytes separately against those in XX oocytes, which were comparable at 0.44 ± 0.14 and 0.48 ± 0.14, respectively. These results indicate that the X-linked DEGs in this category were transcribed from each X chromosome, independent of the presence of another X chromosome or the Y chromosome.

The X chromosome dosage may negatively affect the transcript levels of other genes. A reverse relationship between the X chromosome dosage and transcript levels was found when DEGs were analysed by overlapping among XX < XO, XX < XY, XO > XY, or XO < XY comparison groups with the focus on the loss by the second X chromosome (Supplementary Figure S1). 10 (4.3%) out of total 232 X-linked DEGs and 283 (4.2%) out of 6,801 autosomal DEGs showed lower transcript levels in XX oocytes than XO or XY oocytes without difference between XO and XY oocytes. Examples are *Prps2*, phosphoribosyl pyrophosphate synthetase, and *Tceal8*, transcription elongation factor A. Since no bias towards X-linked DEGs is seen in this category, these results may suggest that the transcript levels of X chromosome dosage dependent DEGs negatively affected other genes including some X-linked genes.

### X-Linked Gene Dosage Compensation

X-linked gene expression in somatic cells is known to be evolutionally adjusted by two mechanisms; 1) the transcript levels are upregulated to match those by autosomal genes in XY cells (gene dosage compensation) and 2) one of the two X chromosomes is inactivated in XX cells to match XY cells (X inactivation) (Nguyen and Disteche, 2006; Deng et al., 2014). Previous studies have reported that the primordial germ cells that have arrived at gonads follow these principles, but the oocytes violate them starting with the X chromosome reactivation prior to the onset of meiosis (Fukuda et al., 2015; Sangrithi et al., 2017). Since the oocytes during the growth phase have not yet been tested for the X-linked gene dosage compensation, we analysed our normalized RNA-Seq data of all genes with detectable transcript levels excluding Y-linked genes. Due to a large variation of reads, we divided all genes evenly into 6 bins according to their transcript levels and calculated the X:A ratio in each bin (Supplementary Figure S2A). The results showed that the X:A ratio was 1.0 in all XX, XO, and XY oocytes when transcript levels were low (Bins 1–3). However, when transcript levels were the highest (Bin 6), the X:A ratio increased to 1.5 in XX oocytes while it remained near 1.1 in both XO and XY oocytes. Similar trend was found to a lesser extent for the genes with the intermediate transcript levels (Bins 4 and 5). Thus, X-linked gene transcript levels were adjusted during oocyte growth in two distinct manners. For a half of genes, which were transcribed at low levels, their transcript levels were upregulated to match those of autosomal genes in XO and XY oocytes (typical X-linked gene dosage compensation) while they were suppressed to match those of autosomal genes in XX oocytes by a mechanism other than X inactivation. By contrast, for another half of X-linked genes, which were transcribed at higher levels, their transcript levels corresponded to the X chromosome dosage.

Similarly to Figure 6, we calculated the ratio of the transcript levels of individual X-linked genes in Bin 6 by dividing the mean levels in XO and XY oocytes by those in XX oocytes (Supplemental Figure S2B). The ratios distributed within 0.58 ± 0.27 (mean ± SD) along the entire X chromosome. The mean increased by 0.1 and SD was doubled compared to the ratio distribution in Figure 6 where only DEGs were analysed. These results indicate that highly transcribed X-linked genes generally follow the X chromosome dosage dependence.

### Y-Linked-Gene Transcription

**FIGURE 7:**
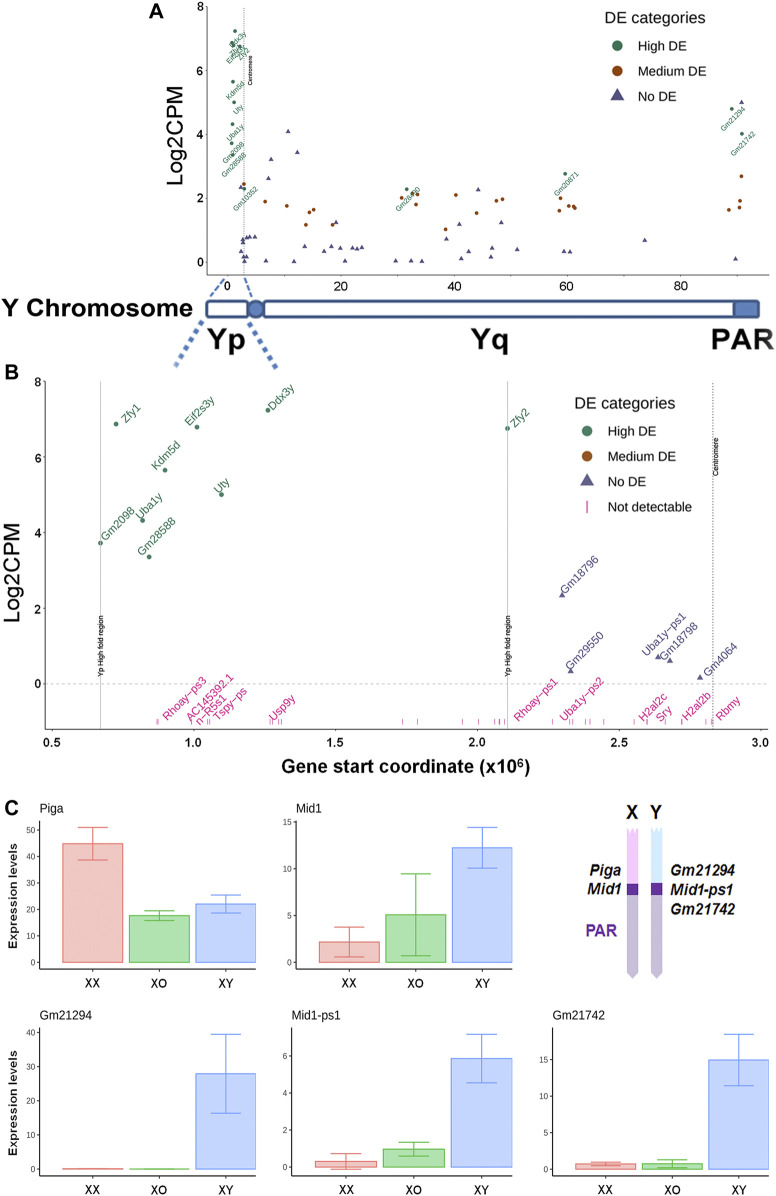
Transcript levels of Y-linked genes in XY oocytes, compared to XX oocytes. **(A)** The entire Y chromosome. High DE, Log2FC ≥ 3. Medium DE, 1 < Log2FC < 3, No DE, Log2FC ≤ 1. **(B)** The Y short arm alone. The X axis indicates the distance from the distal end of Yp. The genes indicated in pink are undetectable. **(C)** Transcript levels of X and Y homologous DEGs near the boundary of pseudoautosomal region (PAR). Geometrical mean ± SD. The diagram **(top, right)** indicates the approximate positions of examined genes on the X and Y chromosomes.

Our XY female mouse model provides a unique opportunity for examining whether and how Y-linked genes are transcribed outside the male germline. From 1,570 reads of Y-linked genes in our data, 39 genes were detected in XY oocytes (Figures 7A,B) while very few reads were detectable near threshold levels in XO or XX oocytes. On the short arm, the nine genes with high transcript levels (Log2FC ≥ 3.0) were clustered within a narrow region except for *Zfy2*. On the long arm, 21 repetitive-sequence-genes, which are not yet well defined, were transcribed at medium levels (1 < Log2FC ≤ 3.0). By contrast, well known genes such as *Sry*, *Usp9y*, and *Rbmy* on the short arm and *Sly* and *Ssty1/2* on the long arm were undetectable in XY oocytes, suggesting that these genes may have been actively repressed. Of the 13 highly transcribed genes, *Gm21294* is proximal to the boundary of pseudoautosomal regions (PAR) on the Y long arm and its transcript levels were detectable only in XY oocytes as expected (Figure 7C). The PAR boundary is located in the intron 3–4 of *Mid1* (*Trim18*) on the X chromosome while its truncated form *Mid1-ps1* as well as *Gm21742* are located within the PAR on the Y chromosome (Palmer et al., 1997; Perry et al., 2001; Lu et al., 2013). In our results, transcript levels of both *Mid1-ps1* and *Gm21742* were much higher in XY oocytes than in XX or XO oocytes (Figure 7C). Transcript levels of X-linked *Mid1* were also higher in XY oocytes than in XX or XO oocytes although the differences were more moderate. These results were unexpected as genes in the PAR should behave like autosomal genes. By contrast, *Piga*, which is proximal to the boundary of the PAR on the X chromosome, was transcribed at higher levels in XX oocytes than XO or XY oocytes, representing the X chromosome dosage dependent X-linked DEGs. Thus, Y-linked genes at the boundary of and within the PAR were more actively transcribed in XY oocytes than in XX or XO oocytes during the growth phase.

### Sex Chromosome Dosage Dependent Differentially Expressed Genes

We next asked whether the XO oocyte has the disadvantage of missing one sex chromosome compared to the XX or XY oocyte which carries two sex chromosomes. Indeed, in the Venn diagram with focus on the gain by the second X chromosome (Figure 5C), 2,681 (28.6%) out of all 9,358 autosomal DEGs showed comparable transcript levels between XX and XY oocytes, but significantly lower in XO oocytes (solid purple circle). In addition, 102 (1.1%) autosomal genes were lower in XY oocytes than in XX oocytes, but still higher than in XO oocytes (broken purple circle). These results may suggest that homologous genes on X and Y chromosomes share similar effects to maintain the transcript levels of many autosomal genes.

**FIGURE 8:**
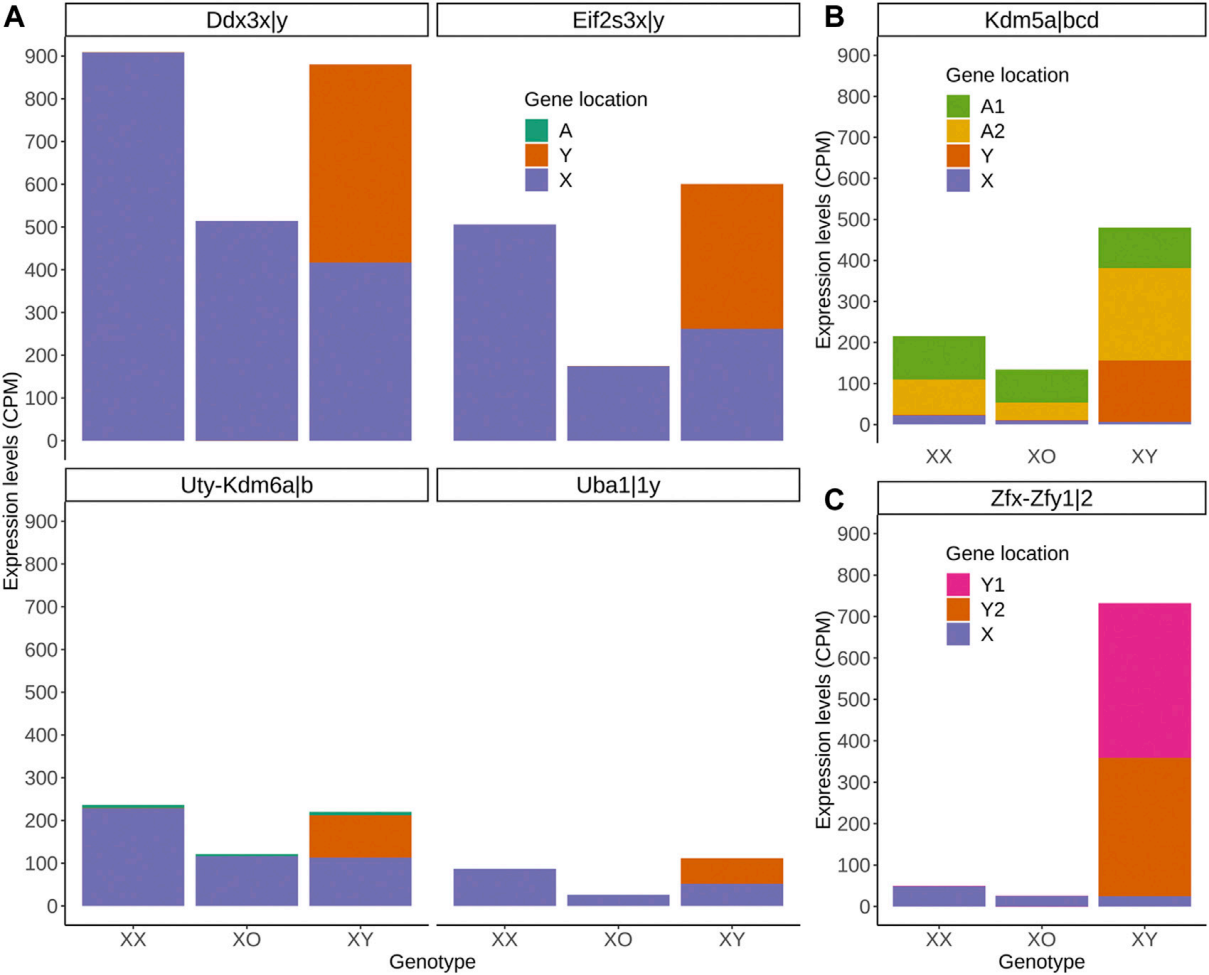
Transcript levels of X-, Y-, and autosomal homologs in XX, XO, and XY oocytes in the growth phase. **(A)** X- and Y-homologs with (*Kdm6*) or without (others) an autosomal homolog. **(B)** X- and Y-homologs (*Kdm5c* and *Kdm5d*, respectively) and two autosomal homologs (A1 = *Kdm5a*, A2 = *Kdm5b*). **(C)** X- (*Zfx*) and two Y-homologs (Y1 = *Zfy1*, Y2 = *Zfy2*).

Of the highly expressed Y-linked genes, *Zfy1/2* is known to have distinct biological activities from their X homolog *Zfx* (Luoh et al., 1997; Shpargel et al., 2012). However, *Ddx3y* shares RNA helicase activity and may be exchangeable with its X homolog *Ddx3x* (Sekiguchi et al., 2004; Matsumura et al., 2019). *Eif2s3y* and *Eif2s3x* also share redundant functions in the proliferation of spermatogonia although they exhibit distinct activities when overexpressed in the ES-derived female germline (Yamauchi et al., 2016; Hamada et al., 2020). *Uty* encodes a protein, which can partially compensate for the embryonic lethality by null mutation of its X-homolog *Utx* (*Kdm6a*) although UTY has a lower histone demethylase activity than UTX (Shpargel et al., 2012; Welstead et al., 2012). These homologs may have contributed to the regulation of sex chromosome dosage dependent autosomal DEGs. To explore this possibility, we compared the transcript levels of X- and Y-homologs, and autosomal homologs if available, in XX, XO and XY oocytes (Figure 8). The results show a consistent trend that transcript levels of X-homologs were comparable between XO and XY oocytes and twice higher in XX oocytes, fitting into the X chromosome dosage dependent X-linked DEGs category. Furthermore, the transcript levels of Y-homologs were comparable with those of X homologs in XY oocytes, making the sum comparable between XX and XY oocytes (Figure 8A). Thus, simple comparison of transcript levels supports our hypothesis that the Y chromosome may compensate for the deficiency of one X chromosome in XY oocytes. One exceptional case is *Kdm5* with lysine-specific demethylase activity, which has an X-homolog (*Kdm5c*), a Y-homolog (*Kdm5d*) and two autosomal homologs (*Kdm5a* and *Kdm5b*). While transcript levels of *Kdm5a* were consistent in the oocytes of all three genotypes, those of *Kdm5b* were much higher in XY oocytes, bringing the sum of all homologs to the highest, compared to XX or XO oocytes. Another exception is the *Zfx/y* pair, where the transcript levels of Y-homologs *Zfy1/2* surpassed those of X-homolog *Zfx*.

### Differentially Expressed Genes in XY Oocytes Compared to XX and XO Oocytes

**FIGURE 9:**
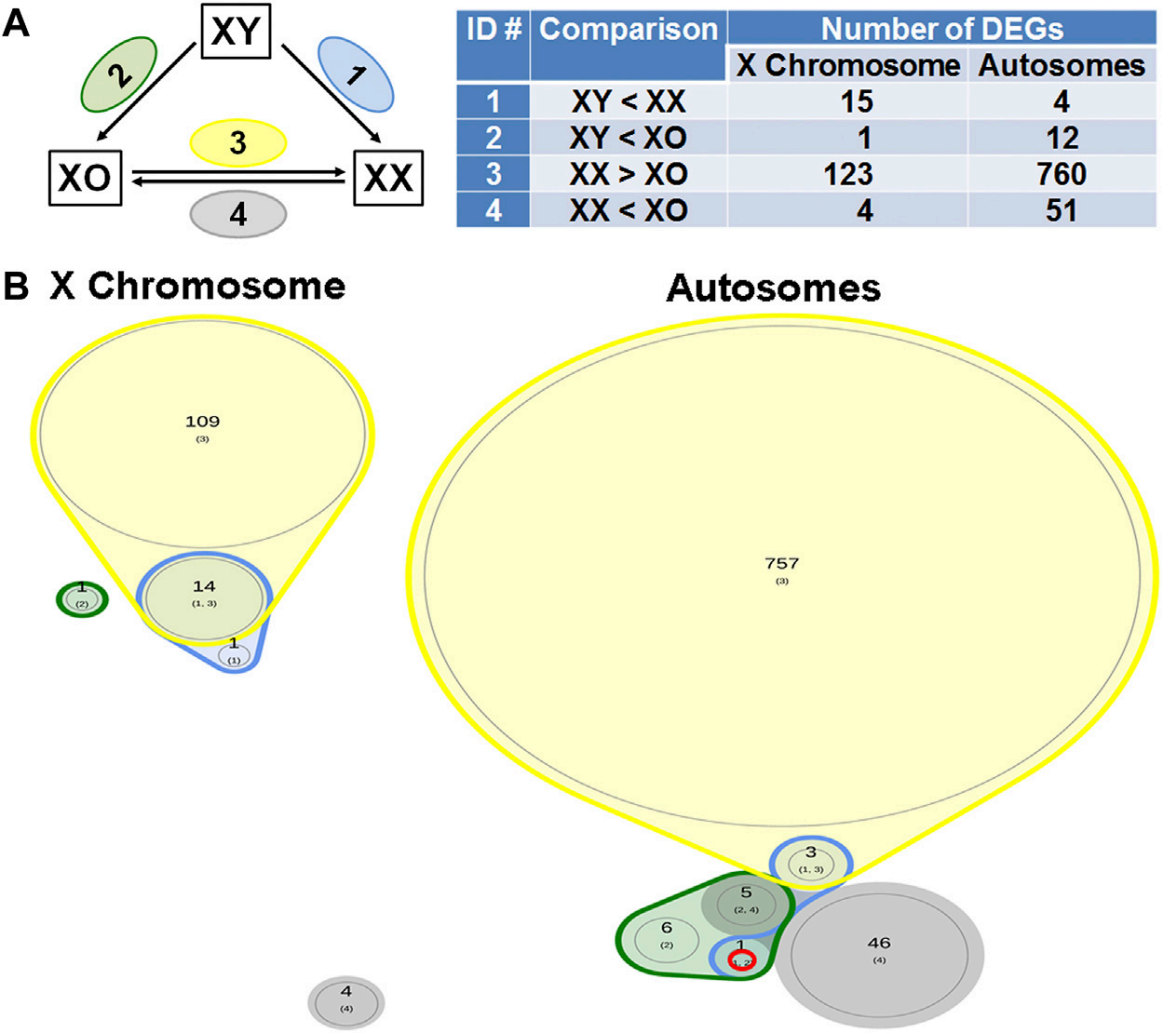
DEGs lower in XY oocytes than in XX or XO oocytes. **(A)** The total number of DEGs (*p* < 0.05, Log2FC > 1) in four comparison groups with focus on loss by the Y chromosome. **(B)** Venn plots to indicate the overlapping of DEGs among four comparison groups. Only one autosomal gene, *Larp6* on Chromosome 9 (ENSMUSG00000034839.5), was found in this category.

**FIGURE 10:**
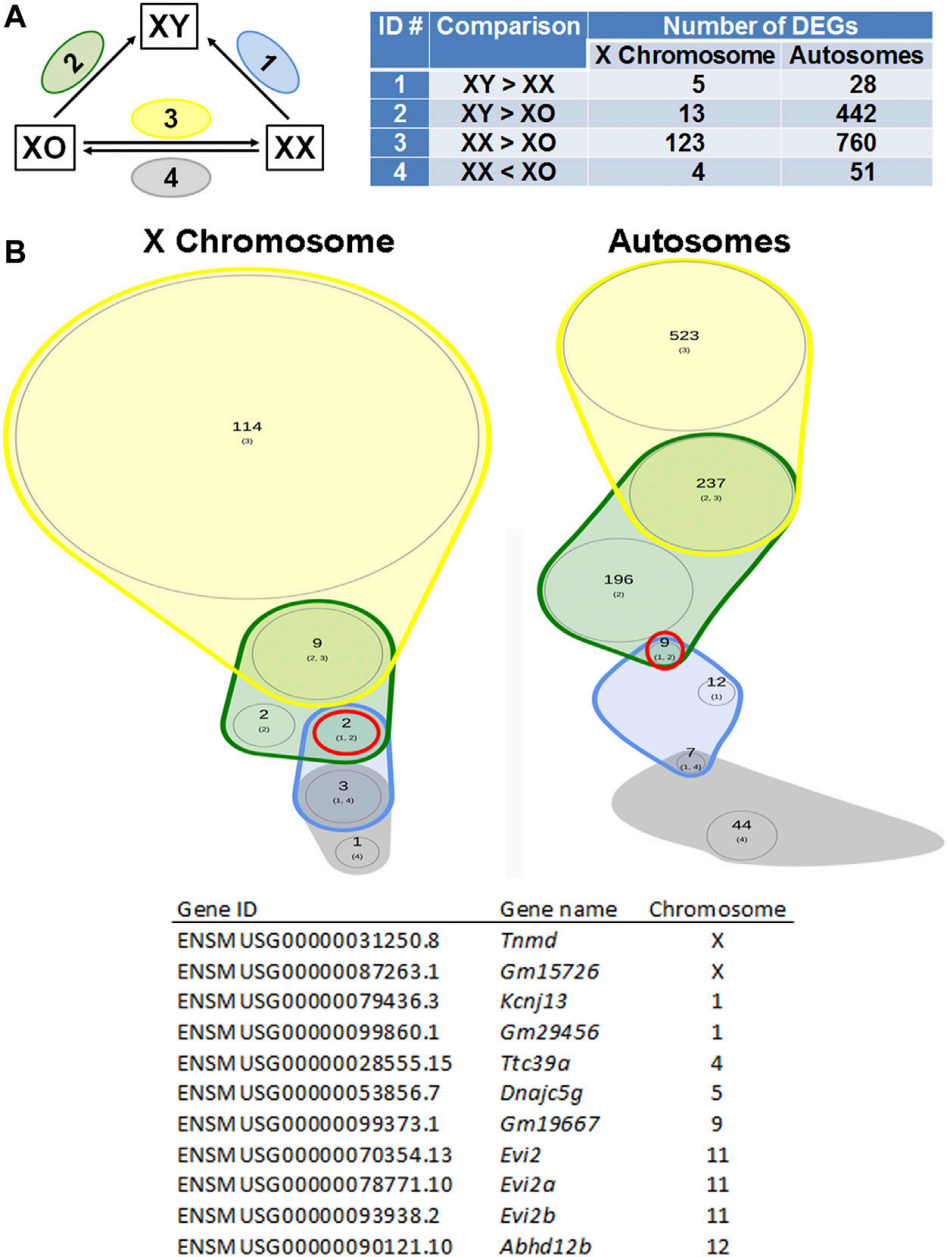
DEGs higher in XY oocytes than in XX or XO oocytes. **(A)** The total number of DEGs (*p* < 0.05, Log2FC > 1) in four comparison groups with focus on gain by the Y chromosome. **(B)** Venn plots to indicate the overlapping of DEGs among four comparison groups. Two X-linked genes and nine autosomal genes were found in this category as listed in the table.

If any gene is responsible for the cytoplasmic defects in XY oocytes, such gene must be differentially expressed compared to both XX and XO oocytes. In the Venn diagram with focus on the loss by the second Y chromosome (Figure 9), none out of 129 X-linked DEGs and only one gene, *Larp6*, out of 818 autosomal DEGs showed lower transcript levels in XY oocytes than in both XX and XO oocytes. *Larp6* encodes a RNA-binding protein with diverse functions including tRNA processing, non-coding RNA metabolism, and ribosomal biogenesis (Maraia et al., 2017) By interrogating the Venn diagram in Figure 5C, the transcript levels of X-linked *Bmp15* also turned out to be the lowest in XY oocytes among the three genotypes. *Bmp15* encodes an oocyte secretary factor that promotes follicular growth and ovulation (Yan et al., 2001; Su et al., 2004; Sugiura et al., 2007). In the reverse relationship, 2 genes, *Tnmd* and *Gm15726*, out of 131 X-linked DEGs and 9 out of 1,028 autosomal DEGs were found at higher levels in XY oocytes than both XX and XO oocytes (Figure 10). X-linked *Tnmd* encodes a type II transmembrane protein, which has potent anti-angiogenic activity (Shukunami et al., 2005). An autosomal gene *Kcnj13* encodes an inwardly rectifying potassium channel (KIR7.1), conserved in vertebrates, and KCNJ13-regulated membrane potential modulates actin organization in tracheal smooth muscle cells (Yin et al., 2018).

### Changes in Gene Transcript Levels in XX, XO, and XY Oocytes During Follicular Growth

To determine when DEGs became differential among XX, XO, and XY oocytes during follicular growth, we compared the transcript levels of selected genes in the oocytes during the entire growth phase as well as at the end of growth phase (FG-oocytes) using quantitative RT-PCR (qRT-PCR). Since oocytes of different sizes were collected from ovaries at different ages for technical necessity, we compared the transcript levels in the oocytes of 40–50 μm at 8 and 12 dpp and those of 50–60 μm at 12 and 18 dpp, but the results were consistent (not shown) and combined.

We first determined Cq values of *Ppia* and *Tubb5*, selected from most consistently highly expressed genes among XX, XO, and XY oocytes of 50–60 µm in the RNA-Seq data, using cDNA aliquots from pooled XY oocytes in biological triplicates (Supplementary Figure S3). Based on relative consistency in the Cq values throughout the oocyte growth, we chose *Ppia* for normalization of Cq values of other genes in each cDNA sample and qPCR amplification.

We then determined the transcript levels of three Y-linked genes, *Ddx3y*, *Uba1y*, and *Zfy1/2*, in XY oocytes (Supplementary Figure S4). *Ddx3y* encodes a protein with characteristics of Dead-box RNA helicase, which is dispensable for male fertility (Matsumura et al., 2019). *Uba1y* (also named *Ube1y*) encodes a protein with homology to a ubiquitin activating enzyme although its function remains unknown (Mazeyrat et al., 2001). *Zfy1* and *Zfy2* encode transcription factors possessing zinc finger domains and an acidic domain (Koopman et al., 1991; Decarpentrie et al., 2012). Because of the highly homologous sequences, we could not design primers to differentially amplify *Zfy1* and *Zfy2*, and the results of qRT-PCR indicate the sum of *Zfy1* and *Zfy2* transcript levels. Our qRT-PCR results showed that the transcript levels of *Ddx3y* and *Uba1y* were low in the oocytes of 20–30 μm, gradually increased with oocyte growth, and peaked in FG-oocytes. The transcript levels of *Zfy1/2* increased slightly in the oocytes from 40 to 60 µm but remained at low levels throughout the growth phase. This is the only case that did not agree with the results of RNA-Seq, in which the transcript levels of both *Zfy1* and *Zfy2* were as high as those of *Ddx3y* and *Uba1y*. Quantitation of transcript levels by RNA-Seq should be more accurate since qRT-PCR is limited by the design of primers.

We selected *Eif2s3x* and *Atrx* to represent X chromosome dosage dependent X-linked DEGs and *Bmp15*, whose transcript levels were further decreased in XY oocytes than XO oocytes, in our RNA-Seq data. *Eif2s3x* encodes a eukaryotic translation initiation factor (Ehrmann et al., 1998) while *Atrx* encodes a helicase of the SWI/SNF2 family of chromatin remodeling proteins and its transcripts are enriched in pericentromeric heterochromatins in pachytene oocytes as well as metaphase spermatogonia (Baumann et al., 2008; Levy et al., 2015; Lovejoy et al., 2020). Our qRT-PCR results showed that the transcript levels of *Eif2s3x* were stable in the oocytes from 20 to 60 µm and then drastically increased with further oocyte growth. The transcript levels of *Bmp15* and *Atrx* were very low in the oocytes of 30–40 µm and gradually increased with further oocyte growth and peaked in FG-oocytes. Transcript levels of all three genes were almost always significantly lower in XY oocytes than in XX oocytes all through the growth phase. Transcript levels in XO oocytes were also lower than those in XX oocytes although significant difference was not always found. At the end of growth phase, transcript levels of both *Bmp15* and *Atrx* in XY oocytes were lower than those in XO oocytes and only 13 and 21% of their respective levels in XX oocytes.

**FIGURE 11:**
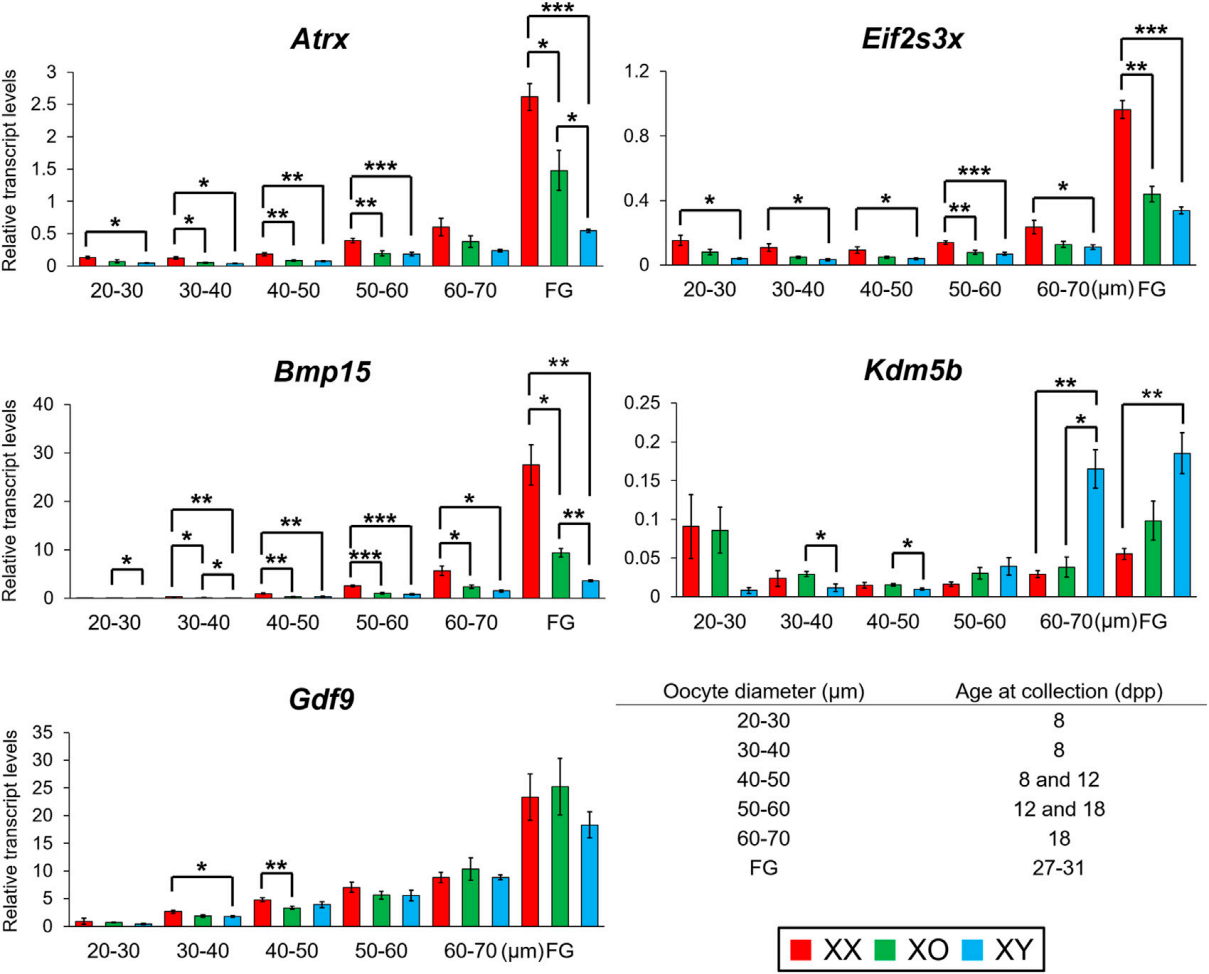
Changes in transcript levels of individual genes in XX, XO and XY oocytes during follicular growth. Transcript levels of X-linked and autosomal genes normalized to *Ppia* transcript levels. *, **, and *** indicate significance at *p* < 0.05, 0.01, and 0.001, respectively, among XX, XO, and XY oocytes of the same diameter by *t*-test.

We chose *Kdm5b* as an autosomal gene whose transcript levels were higher in XY oocytes than XX or XO oocytes in the RNA-Seq data. *Kdm5b* (*Jarid1b*) encodes a histone demethylase of H3K4 involved in gene transcriptional activation (Zhang et al., 2016). *Kdm5b* is particularly interesting as it was the dominant transcripts among *Kdm5* homologs in the oocytes of growth phase, and specifically affected by the XY chromosomal complement (Figure 8). Five alternative splicing isoforms including up to 27 exons have been reported for *Kdm5b* (ENSMUSG00000042207.17) (Supplementary Figure S5A). We designed three sets of primers to cover the junctions of Exons 1–2, 13–14, and 26–27. Only the amplicon of Exons 26–27 was detectable in FG-oocytes while all sets of primers yielded amplicons of expected sizes in the total RNA from ovary and testis (Supplementary Figures S5B,C). In fact, the entire RNA-Seq data of *Kdm5b* showed consistent transcript levels corresponding only to Exons 23–27 in the oocytes of 50–60 µm (Supplementary Figures S5B). Our qRT-PCR results revealed that *Kdm5b* transcript levels were detectable at moderate levels in XX and XO oocytes while barely detectable in XY oocytes of 20–30 µm (Figure 11). *Kdm5b* transcript levels then decreased in both XX and XO oocytes and remained low in the oocytes of all genotypes until the levels in XY oocytes drastically increased to be significantly higher than those in XX or XO oocytes of 60–70 µm. No more difference was found between XO and XY FG-oocytes as the transcript levels in XY oocytes did not increase as much as in XO oocytes at the end of growth phase.

We selected *Gdf9* as a gene whose transcript levels were not different among XX, XO, and XY oocytes of 50–60 µm in the RNA-Seq data. *Gdf9* encodes a TGFβ superfamily member, known to be secreted by the oocyte to facilitate follicular growth beyond the primary follicle stage (Dong et al., 1996). Our qRT-PCR results (Figure 11) show that *Gdf9* transcript levels increased with oocyte growth to peak in FG-oocytes. Minor differences were recognized in the oocytes of small sizes, but no more difference was found among XX, XO, and XY oocytes of 50 µm or larger, in consistent with the RNA-Seq results.

### Enrichment of H3K4me3 in the Nuclei of XX and XY Oocytes

**FIGURE 12:**
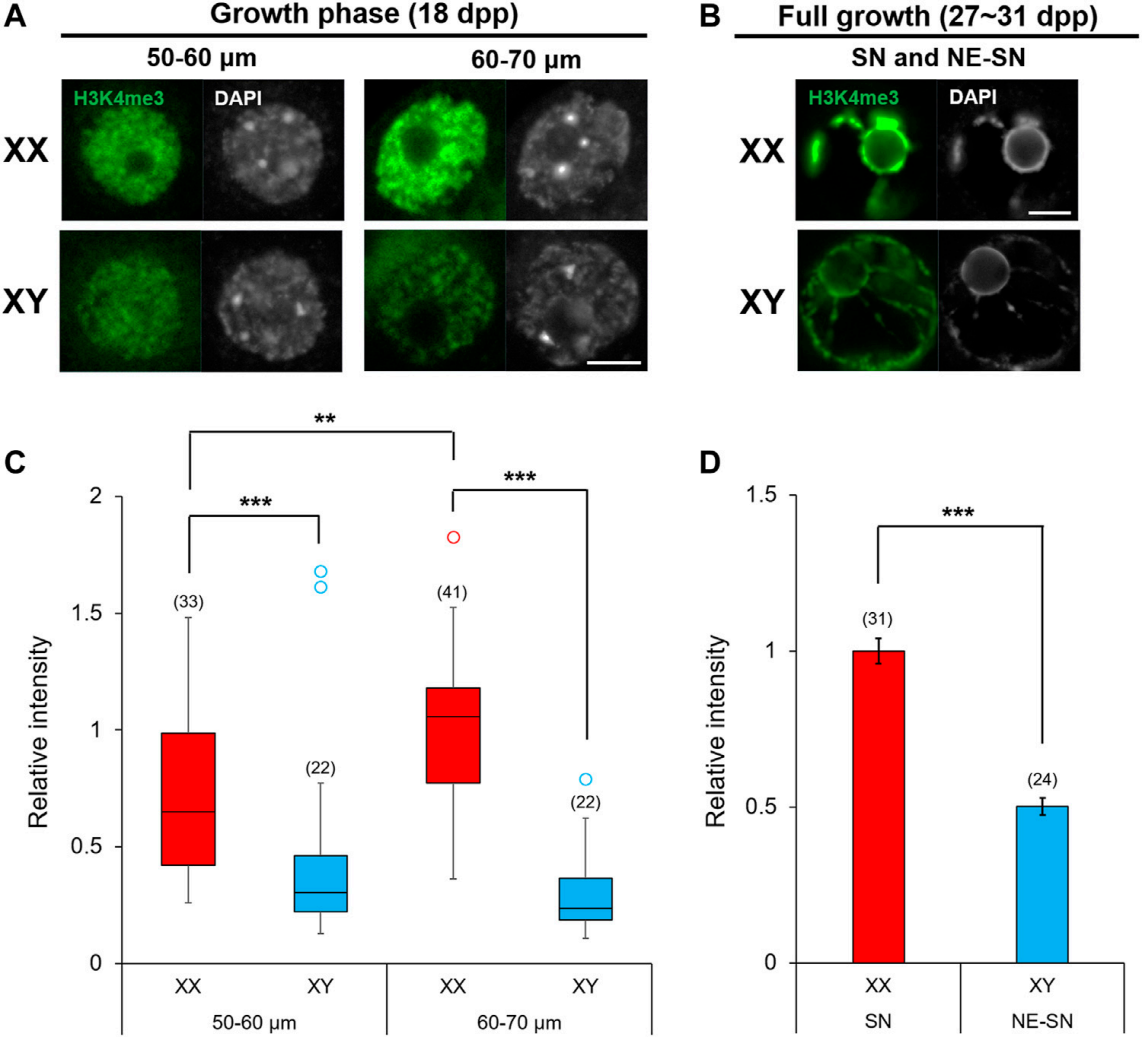
Enrichment of H3K4me3 in XX and XY oocytes in the growth phase. **(A,B)** H3K4me3 immunoflourescence staining in the nuclei of oocytes in the late growth phase and at full growth with DAPI counterstaining. Scale bar, 10 μm. **(C)** Relative intensity of H3K4me3 staining in the oocytes of growth phase. Each box plot indicates the median with 1st and 3rd quartiles. The thin vertical line indicates the range from minimum to maximum values. ° indicates outlier. The total number of oocytes examined is given above each column. ** and *** indicate statistical differences at *p* < 0.01 and 0.001, respectively, by Mann-Whitney U test. **(D)** Relative intensity of H3K4me3 staining in FG-oocytes. Each column indicates the mean ± SEM. The number of oocytes examined is given above each column. *** indicates statistical difference at *p* > 0.001, by Welch’s *t*-test.

Oocytes undergo dynamic epigenetic modifications of histones as well as *de novo* DNA methylation during the growth phase (Lawrence et al., 2016; Gahurova et al., 2017; Stäubli and Peters, 2021). Methylation of specific lysine on histone H3 within a gene domain is associated with gene transcription, chromatin configuration and ultimately the competence of oocytes for embryonic development. For example, methyltransferase SETD2 is crucial for H3K36 trimethylation (H3K36me3) and consequent *de novo* DNA methylation while suppressing ectopic H3K4me3 and H3K27me3 during the oocyte growth (Xu et al., 2019). H3K4me3 in the gene promoter is generally associated with active transcription while H3K27me3 is associated with transcription repression and H3K9me3 causes constitutive heterochromatinization and gene silencing (Puschendorf et al., 2008). Furthermore, EHMT2 (G9A/GLP) is responsible for H3K9me2 affecting chromatin organization in oocytes (Yeung et al., 2019). The methylation status of each site is set on by the balancing act between lysine-specific methyltransferases and demethylases. We have already shown that *Kdm5b* encoding H3K4me3 demethylase was highly expressed in XY oocytes compared to XO or XX oocytes (Figures 8, 11). We interrogated other genes known to be involved in histone modifications, but none was found among the DEGs in our RNA-Seq data. To examine whether the higher transcript levels of *Kdm5b* in XY oocytes reflected into histone modifications, we immunofluorescence-stained for H3K4me3 in XX and XY oocytes of 50–70 µm and at the FG-stage (Figure 12). The results showed that H3K4me3 was enriched in patches over the nuclei of XX and XY oocytes in the growth phase (Figure 12A). H3K4me3 staining intensity on average in XY oocytes was significantly lower than that in XX oocytes of 50–60 or 60–70 µm. Furthermore, H3K4me3 staining intensity increased in XX oocytes from 50–60 to 60–70 µm while it failed to do so in XY oocytes (Figure 12C). In both XX and XY FG-oocytes, strong H3K4me3 signals were observed along the condensed chromatin around the nucleuolus (Figure 12B). However, the total H3K4me3 signals over the nucleus was significantly lower in XY FG-oocytes than in XX FG-oocyte (Figure 12D). These results indicate that the higher transcript levels of *Kdm5b* were associated with lower H3K4me3 accumulation in the XY oocyte nucleus in the late growth phase.

## Discussion

An oocyte accumulates cytoplasmic components such as mRNAs and proteins during follicular growth to support the subsequent meiotic progression, fertilization, and early embryonic development in the absence of *de novo* transcription. However, how gene transcription is regulated during oocyte growth is not well understood. Our current study focused on the transcript levels of X- and Y-linked genes and their association with the genome-wide transcriptome in oocytes. The XY oocyte became abnormal in chromatin configuration, mitochondria distribution and the global *de novo* transcription near the end of growth phase. Therefore, we compared transcriptome among XX, XO and XY oocytes of 50–60 μm, where no difference was yet apparent in their morphology or *de novo* transcription activity. Our results revealed that the X chromosome dosage exerted dominant effects on the transcriptome in XO oocytes, whereas the presence of the Y chromosome or transcripts of Y-linked genes made the transcriptome in XY oocytes closer to that in XX oocytes. Nonetheless, XY oocytes established a distinct transcriptome at the end of growth phase.

### RNA-Sequencing Methods Used in This Study

To accommodate the limited quantity of RNAs from 30 oocytes per sample, we used Smart-seq3 protocol (Hagemann-Jensen et al., 2020). Version 2 of the protocol was already known to work well for single cells for a standard amount of 2 pg total RNA (Picelli et al., 2014). The new version of the protocol features a higher specificity and sensitivity by using a 5′UMI. The standard oligo-dTVN and Template Switching Oligo (TSO) primers were modified, enlarging from 8 to 12N UMIs, which accommodated a relatively larger than single-cell library complexity of our small bulk samples. Besides the existing 5′UMI which was used for our present data analysis, we introduced a 3′UMI in order to be able to keep compatibility with other single-cell quantification methods like 10xGenomics or our previous Nanopore data (Oikonomopoulos et al., 2016). The 3′ end can also be sequenced by using same Nextera Tn5 binding site. Sequencing of both ends allowed for accurate PCR primer design of maximally spanning isoforms, being able to match both transcription start and end sites (TSS/TES).

### Transcription of X-Linked Genes in XX, XO, and XY Oocytes

X and Y chromosomes have evolved from a pair of ancestral autosomes associated with the acquisition of the *SRY* male determining gene on the Y chromosome in mammals (Nagai, 2001; Wallis et al., 2008). While the X chromosome has retained most of its ancestral genes, the Y chromosome has lost most (Soh et al., 2014). This loss of ancestral genes from the Y chromosome led to a disparity in the dosage of X-linked versus autosomal genes between the two sexes and rendered compensation in two ways during evolution. While X-linked genes get upregulated to balance the output of X-linked and autosomal genes in XY somatic cells (X-linked gene dosage compensation), one of the two X chromosomes is inactivated (X inactivation) to balance the output of X-linked genes in XX somatic cells (Deng et al., 2013). However, neither mechanism operates in the germline (Fukuda et al., 2015; Sangrithi et al., 2017) (this study). In the mouse, when the germ cells arrive at the gonadal primordia and initiate sexual differentiation, they display both X-linked gene dosage compensation and X chromosome inactivation like somatic cell lines. Therefore, the X:A ratio is 1.0 in all XX, XO, and XY oogonia. However, when the inactive X chromosome is reactivated in XX oocytes at the onset of meiosis, the X:A ratio transiently increases to 1.7 and then declines to 1.0 in neonatal ovaries, while that in XO oocytes declines to 0.5. Since the two X chromosomes remain transcriptionally active in XX oocytes, transcript levels of X-linked genes appear to be adjusted to those of autosomal genes by suppressing the transcription of both X chromosomes. Such manner of sex chromosome dosage compensation is known in human preimplantation embryos as well as non-mammalian species such as *Drosophila* and *C. elegans* (Parkhurst and Meneely, 1994; Charlesworth, 1996; Soh et al., 2014; Petropoulos et al., 2016).

Our current results revealed two types of X-linked gene dosage compensation in the oocytes of the growth phase. For the genes with low transcript levels, the X:A ratio remained 1.0 in XX oocytes while it was increased to 1.0 in XO and XY oocytes. It appears that the X-linked gene dosage compensation is transiently lost in fetal and neonatal oocytes, but it is resumed in XO and XY oocytes during the resting stage or upon the entry into the growth phase. For the genes with high transcript levels, however, the X:A ratio increased to match the double X chromosome dosage in XX oocytes. Our RNA-Seq results identified 180 X-linked genes at lower transcript levels in XO or XY oocytes than XX oocytes with their ratios clustered around 0.47 along the entire X chromosome. Our qRT-PCR results revealed that *Eif2s3x*, *Bmp15*, and *Atrx* genes maintained the X chromosome dosage dependence during the entire oocyte growth. Thus, some X-linked genes appeared to be relieved from repression to enhance their transcription in XX oocytes during follicular growth. While XO oocytes maintained a half dosage of X-linked genes compared to XX oocytes until the end of growth phase, transcript levels of *Bmp15* and *Atrx* did not increase in XY oocytes as much as XO oocytes. The premature termination of *de novo* transcription in XY oocytes may have contributed to this difference. Alternatively, since FG-oocytes have a unique mechanism to store mRNAs, XY oocytes may have a limited capacity in such a storage mechanism.

### Transcription of Y-Linked Genes in XY oocytes

Mammalian Y chromosomes used to be considered heterochromatic and gene poor. However, the male-specific region of the Y chromosome (MSY) of the B6 mouse strain has now been fully sequenced and turned out to be 99.9% euchromatic and encodes about 700 proteins (Soh et al., 2014). The mouse MSY retains only 2% of ancestral genes while all but 45 of the MSY’s genes were newly acquired and massively amplified during evolution, and consist of three major Y gene families, *Sly* and *Srsy* and *Ssty* (Bishop and Hatat, 1987; Prado et al., 1992). These Y gene families have X homologs that were also newly acquired and amplified on the X chromosome (Soh et al., 2014). Of the ancestral genes, a 1.6 Mb region on the short arm contains seven single-copy genes, *Ddx3y*, *Eif2s3y*, *Kdm5d*, *Uba1y*, *Uty*, *Usp9y*, *Sry* and a pair of duplicated genes, *Zfy1* and *Zfy2* (Bellott et al., 2014). The remaining 0.4 Mb is ampliconic and contains one gene family *Rbmy*. Many of these ancestral genes are ubiquitously expressed, consistent with the speculation that surviving ancestral genes are widely expressed and regulate dosage-sensitive gene expression (Lahn and Page, 1997; Bellott et al., 2014). In our study, all these genes except for *Usp9y*, *Sry*, and *Rbmy* were transcribed at high levels in the XY oocytes of 50–60 µm. By contrast, the newly acquired ampliconic gene families on the long arm, *Sly*, *Ssty*, and *Srsy*, which are reported to be expressed predominantly in the male germline (Toure et al., 2004; Reynard and Turner, 2009), were undetectable in XY oocytes. However, other newly identified but poorly characterized genes on the Y long arm were transcribed at moderate levels in XY oocytes. These genes may be allowed for expression due to a lack of repressive regulators in oocytes. It remains to be determined whether Y-linked genes on the long arm exert biological activities in the oocyte.

*Zfy2* was a candidate Y-linked gene for making the XY oocyte incompetent for embryonic development (Vernet et al., 2016). Both *Zfy1* and *Zfy2* are known to play critical roles in spermatogenesis and spermiogenesis (Vernet et al., 2014a; Vernet et al., 2016; Nakasuji et al., 2017). Furthermore, expression of exogenous *Zfy2*, but not *Zfy1*, in a XO female mouse on a mixed genetic background makes their oocytes incompetent for embryonic development (Vernet et al., 2014b). However, these studies used mice carrying the *Mus musculus molossinu*-type *Zfy2*, which harbors an 18 bp deletion compared to *Zfy1* (Nagamine et al., 1990). By contrast, *Mus musculus domesticus*-type *Zfy2* in the XY female used in this study harbors no such deletion, and none of the minor polymorphic differences (one or two consecutive nucleotide changes) between *Zfy1* and *Zfy2* would contribute to a functional difference. In our RNA-Seq data, both *Zfy1* and *Zfy2* were highly expressed in XY oocytes of 50–60 µm. However, qRT-PCR results showed rather low *Zfy1*/*Zfy2* transcript levels throughout the oocyte growth. Our current results do not exclude but do cast doubt on the contribution of *Zfy2* to the cytoplasmic defects of XY oocytes carrying the Y^TIR^ chromosome.

### Sex Chromosome Dosage Dependent DEGs

We presumed that XY oocytes are inferior to XO oocytes because of the presence of the ectopic Y chromosome and their failure to support embryonic development. However, our current results do not concur with this simplistic prediction. The transcript levels of the one thirds of autosomal DEGs were comparable between XX and XY oocytes but significantly lower in XO oocytes of 50–60 µm. XO oocytes appear to accumulate sufficient mRNAs by the end of growth phase to become competent for embryonic development. Nonetheless, the haplo-insufficiency of many gene products during the growth phase may make XO oocytes more vulnerable to genetic manipulations than XX oocytes. As discussed above, expression of a *Zfy2* transgene causes infertility in XO but not in XX female mice (Vernet et al., 2014b). Our current results show that the transcriptome landscape in XO oocytes is far off that in XX oocytes, more so than in XY oocytes of 50–60 µm. We predicted that the haplo-deficiency in essential X-linked genes was compensated by their Y-linked homologs to make XY oocytes closer to XX oocytes. In general, transcript levels of X homologs corresponded to the X chromosome dosage while those of Y homologs raised the sum in XY oocytes to match that in XX oocytes. Whether X and Y homologs share biological activities with comparable potencies in oocytes remain to be tested.

An interesting case is the genes in the PAR of sex chromosomes. In theory, these genes must behave like autosomal genes. However, two Y-linked genes within the PAR (*Gm21742* and *Mid1-ps1*) and one X-linked gene at the PAR boundary (*Mid1*) were transcribed at much higher levels in XY oocytes than in XX oocytes of 50–60 µm. Their transcript levels in XO oocytes were slightly higher than in XX oocytes, but still much lower than in XY oocytes. Thus, some genes within the PAR did not act like autosomal genes in oocytes. This phenomenon appears to be unique to the PAR since X-linked *Piga* and Y-linked *Gm21294* proximal to the PAR were transcribed as expected; X chromosome dosage dependent and XY specific, respectively. We speculate that the higher order structure or position of the Y chromosome in the XY oocyte nucleus may have favored high transcript levels of genes in the PAR.

### Oocyte-Specific Alternative Splicing of *Kdm5b*

Most of multiexon-containing genes are subject to alternatively splicing. RNA-Seq data provide a great opportunity to identify oocyte-specific splicing isoforms. In our current study, we found such an example for *Kdm5b*. KDM5B, which catalyzes H3K4 demethylation, is presumed to be a transcriptional repressor since H3K4me3 is enriched at transcriptional start sites of active genes (Santos-Rosa et al., 2002; Schneider et al., 2004). However, the role of KDM5B in gene transcription is more complex and depends on cell type and stage. For example, depletion of KDM5B leads to lower H3K4me3 levels in promoter regions and higher H3K4me3 levels in gene body regions in mouse embryonic stem cells (He and Kidder, 2017). Consequently, KDM5B plays an integral role in regulating RNA Polymesase II occupancy, transcriptional initiation and elongation, and alternative splicing of target genes. In our current study, we found a novel oocyte-specific *Kdm5b* isoform, which was upregulated in XY oocytes near the end of growth phase. This isoform includes the catalytic domain and has the potential to exert the demethylase activity. The results with immunofluorescence staining indicated that the higher levels of *Kdm5b* transcript levels reflected into lower enrichment of H3K4me3, the target of KDM5B, in the nucleus of XY oocytes compared to XX oocytes during the growth phase.

### Genes That Are Not Differentially Expressed

It is important to note that some genes which are known to play critical roles in the development of oocyte competence were not differentially expressed among XX, XO, and XY oocytes in our current study. Examples are *Gdf9* and *Tgf-β*, two oocyte secretory factors that are essential for proliferation, differentiation, and metabolism of surrounding follicular cells; *Gja1*, *Gja4*, *Cdh1*, *Cdh2*, and *Ptk2*, which are involved in the oocyte-granulosa cell communication; and *Zfp36l2*, which is important for global transcriptional silencing. TBP2, a TATA binding protein, is a central basal transcriptional regulator that drives cell type-specific features in the oocyte and is known to upregulate both *Gdf9* and *Bmp15* (Gazdag et al., 2009). However, we did not find a difference in the *Tbp2* transcript levels among XX, XO and XY oocytes, suggesting that the transcript levels of *Brmp15* were lower in XY oocytes due to the X chromosome dosage rather than availability of transcription factors. Finally, other than *Kdm5b*, none of the genes involved in post-translational histone modifications or DNA methylation were among the DEGs. Such results are consistent with our previous finding that the nucleus of XY FG-oocytes can generate healthy pups after its transfer into the XX oocyte cytoplasm (Obata et al., 2008).

### Abnormal Morphological and Transcriptional Features in XY Oocytes at the End Of Growth Phase

Near the end of growth phase, XY oocytes exhibited abnormal chromatin configuration and mitochondria distribution, associated with premature silencing of *de novo* transcription. We speculate that all the morphological abnormalities observed in XY FG-oocytes are due to the altered gene transcription during the growth phase. We cannot pinpoint a gene or genes in this effect. However, X-linked *Bmp15* particularly attracted our attention. Its transcript levels initially corresponded to the X chromosome dosage, however, while they rapidly increased in XX oocytes with further growth, they increased much slowly in XY oocytes and their difference reached 9:1 at the end of growth phase. *Bmp15* encodes a TGFβ family member, known to be secreted by the oocyte to promote proliferation and glucose metabolism in the neighboring granulosa cells (Yan et al., 2001; Su et al., 2004; Sugiura et al., 2007). Since oocytes do not express the glucose transporter, they rely on the supply from the granulosa cells of glycolysis intermediates such as lactate and pyruvate for ATP production by mitochondria (Su et al., 2004; Su et al., 2008; Sutton-McDowall et al., 2010). Glucose metabolism through the pentose phosphate pathway is also crucial for the antioxidant defense and nucleotides supply in the oocyte (Richani et al., 2021). Our previous study has indeed shown that XY FG-oocytes produce lesser amounts of paracrine factors that regulate glycolytic gene expression in its companion granulosa cells, resulting in lower ATP contents in both granulosa cells and the enclosed oocyte (Xu et al., 2014). It is possible that low BMP15 levels indirectly affected global metabolism and gene transcription in XY oocytes during the growth phase.

*Larp6* was the only gene to be found with lower transcript levels in XY oocytes compared to XX and XO oocytes of 50–60 µm in our RNA-Seq data. LARPs are a family of evolutionarily conserved RNA-binding proteins with diverse functions including tRNA processing, non-coding RNA metabolism, ribosomal biogenesis and mRNA translation (Maraia et al., 2017). Therefore, the low LARP6 levels may have contributed to the premature silencing of global gene transcription in XY oocytes. LAPR6 is also known to play a key role in mRNA translocation and translation at the protrusion of mesenchymal cells in culture (Dermit et al., 2020). Although the oocyte does not protrude, its surrounding follicular cells send numerous transzonal projections to the oocyte for exchanging molecules and metabolites (El-Hayek et al., 2018). It would be an attractive possibility that low *Larp* transcript levels are associated with poor communication with follicular cells and consequent lower metabolic supply.

By contrast, X-linked *Tnmd* was found to be transcribed at higher levels in XY oocytes than XO and XX oocytes in our RNA-Seq data. *Tnmd* encodes a type II transmembrane protein, which has potent anti-angiogenic activity necessary for cartilage and retina differentiation (Shukunami et al., 2005). *TNMD* is also highly expressed in human adipose tissue and involved in insulin resistance and glucose metabolism (Senol-Cosar et al., 2016). Altered expression of this gene may also have indicated abnormal metabolism in XY oocytes.

The unusual chromatin configuration in XY FG-oocytes puzzled us. It is known that the transition from NSN- to SN-type chromatin configuration is accompanied by a dynamic relocation of centromeres towards close apposition with the perinucleolar heterochromatin rim (de la Fuente et al., 2004a; Bonnet-Garnier et al., 2012). However, in addition to SN- and PSN-type condensation, XY oocytes exhibited chromatin condensation along the nuclear envelop, which was never seen in XX or XO oocytes. It has been reported that the perinucleolar centromere accumulation involves chromatin remodeling proteins such as ATRX (de la Fuente et al., 2004b). ATRX may have contributed to the abnormal chromatin configuration in XY oocytes since *Atrx* transcript levels in XY oocytes were lower than in XX oocytes throughout the growth phase and even lower (21%) at the end of growth phase. Lower ATRX accumulation may have allowed for the transcription of repetitive sequences such as transposable elements, which are normally repressed in oocytes. However, no ectopic expression or upregulation of genes near centromeres was found in XY oocytes in our RNA-Seq data.

The abnormal chromatin condensation along the nuclear envelop was frequently, but not always, accompanied by accumulation of mitochondria with high membrane potential surrounding the nucleus in XY FG-oocytes. For comparison, mitochondria were homogeneously distributed across the cytoplasm in XX and XO oocytes in agreement with previous reports (Dumollard et al., 2004; Al-Zubaidi et al., 2019). In our RNA-Seq data, none of mitochondrial genes were included in the DEGs in XY oocytes vs. XX and XO oocytes. However, mitochondrial function depends on the coordination of mitochondrial and nuclear genomes, the latter of which encode ∼1,000 proteins and have not yet been thoroughly interrogated in our study. Mitochondrial activity, mediated by ATP production, affects histone modifications and transcription, and ultimately the developmental competence of oocytes (Van Blerkom, 2011; Matilainen et al., 2017). While all XY oocytes exhibited abnormal chromatin configuration, fewer oocytes showed abnormal mitochondrial distribution. Therefore, the altered mitochondrial distribution is more likely a consequence of other metabolic defects in XY oocytes. Nonetheless, further studies are needed to establish the hierarchical relationship between differential gene transcription, epigenetic modification, chromatin configuration and mitochondrial distribution, all of which appeared to be interrupted in the XY oocytes near the end of growth phase.

## Data Availability

The sequencing data, as well as full length coverage tracks, raw 5’UMI, and normalized expression matrices reported in this study have been deposited in the Gene Expression Omnibus website with accession code GSE184153.
