# Supplementary material for: Effects of the Sex Chromosome Complement, XX, XO, or XY, on the Transcriptome and Development of Mouse Oocytes During Follicular Growth
Source: Front Genet. 2021 Dec 20;12:792604. doi: 10.3389/fgene.2021.792604 (PMC8721172; doi:10.3389/fgene.2021.792604)
Supplement: Supplementary file 1 [file Image1.pdf]

Supplemental Materials

Effects of the sex chromosome complement, XX, XO or XY, on the transcriptome and development of mouse oocytes during follicular growth

Wataru Yamazaki, Dunarel Badescu, Seang Lin Tan, Jiannis Ragoussis and Teruko Taketo

Table S1: Primers for qRT-PCR

Figure S1: Reverse X chromosome dosage dependent differentially expressed genes

Figure S2: Ratio of the average transcript levels of all highly expressed X-linked genes in XO and XY oocytes against those in XX oocytes

Figure S3: Changes in transcript levels of *Ppia* and *Tubb5* in XY oocytes during follicular growth.

Figure S4: Changes in transcript levels of Y-linked genes in XY oocytes during follicular growth.

Figure S5: *Kdm5b* transcripts analyses in the oocytes of the growth phase.

Table S1. Primers for qRT-PCR

Gene	Accession number	Primer sequences (5'-3')	Annealing temperature (°C)	Amplicon length (bp)
Ppia	NM_008907.2	CCACCGTGTTCTTCGACATCA<br>CACCACCCTGGCACATGAA	58~62	186
Tubb5	NM_011655.5	GGCAACCAGATCGGTGCTA<br>TGAGCGAACGGAGTCCATAG	58	198
Ddx3y	NM_012008.2	CGCACAGGACGTGTAGGAAA<br>GAACGTCCACGGCTACTTCC	61	173
Uba1y	NM_001357043.1	CAGGAGCTGCAGAGTACCAG<br>AAGCAGCTTGTCTGGAGTAGG	58	90
Zfy1/2	Zfy1: NM_009570.4<br>Zfy2: NM_009571.2	TGCCAGAATCAACTGTGAGGA<br>TGCATTCATGGTAACTTCAGAGGA	58	94
Bmp15	NM_009757.5	ACACAGTAAGGCCTCCCAGA<br>GGTAAACCACAGTGGCTCTGA	58	105
Eif2s3x	NM_012010.3	GTGAGGGTGGAGTGACTCTCG<br>CCATGAGCTACGTGACCAATTGTA	61	148
Atrx	NM_009530.2	TTGTGGACAGCAGGTCAATCA<br>ATCCATTCCATCTGAGTCACGG	62	133
Kdm5b<br>Exon 1-2		CGAGCTGGGAAGAGTTCGC<br>GACATCACAAGCGAATGGTGG	61	125
Exon 13-14	NM_152895.2	CATGATCGAGGACGAGAAAGC<br>GCTCATCATCTGGCAACAGC	61	98
Exon 26-27		AAACTGAGTCACCCCAAGGA<br>GACCCAGTCCACCTCATCTC	58	195
Gdf9	NM_008110.2	ACAATACCGTCCGGCTCTTC<br>AGTGTTCCATGGCAGTCACC	60	126

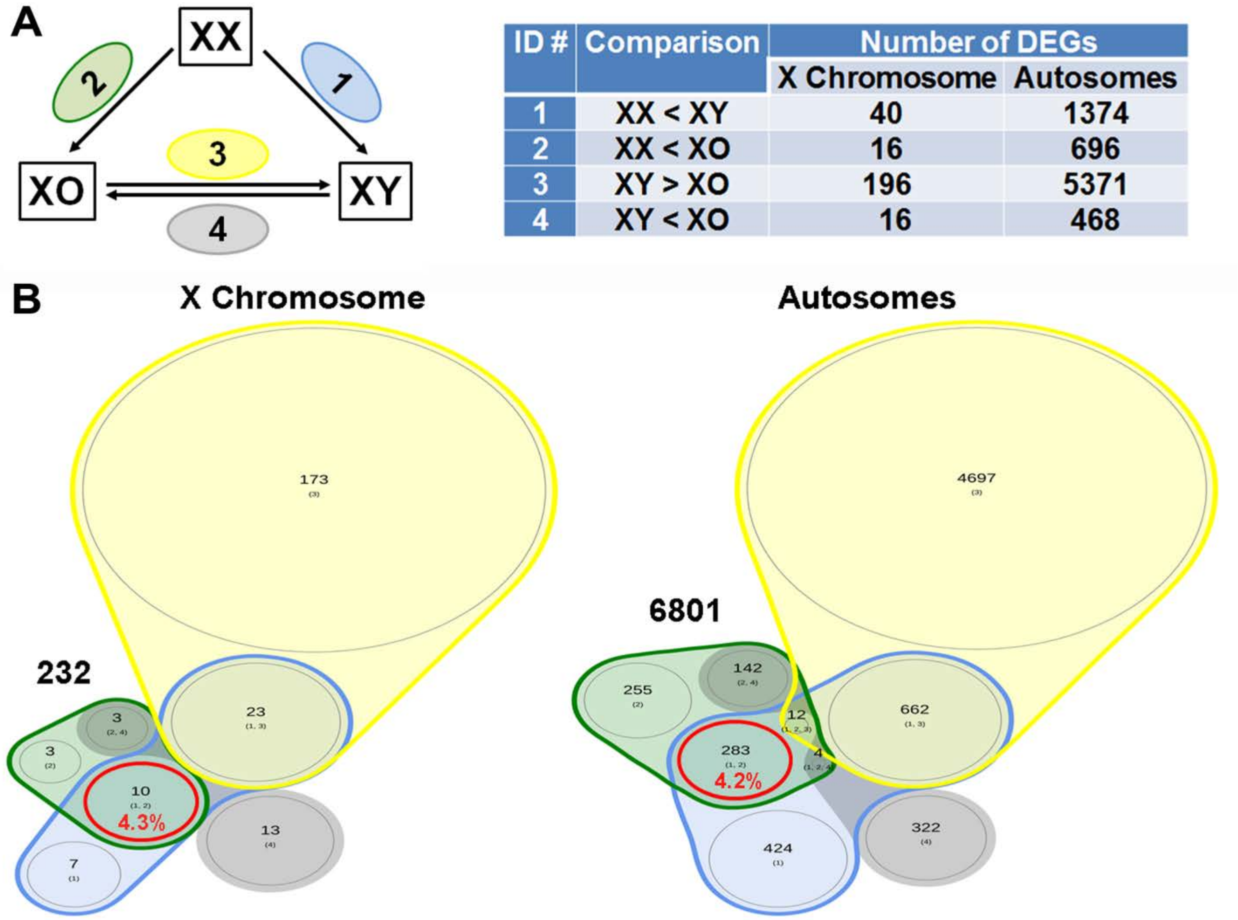

ID #	Comparison	Number of DEGs	
		X Chromosome	Autosomes
1	XX < XY	40	1374
2	XX < XO	16	696
3	XY > XO	196	5371
4	XY < XO	16	468

Figure S1. Reverse X chromosome dosage dependent differentially expressed genes. **A** The total number of DEGs (P<0.05) in four comparison groups with focus on loss by the second X chromosome. **B** Venn diagrams to indicate the overlapping of DEGs among four comparison groups. The red circle indicates DEGs lower in XX oocytes than XO and XY oocytes without difference between XO and XY oocytes.

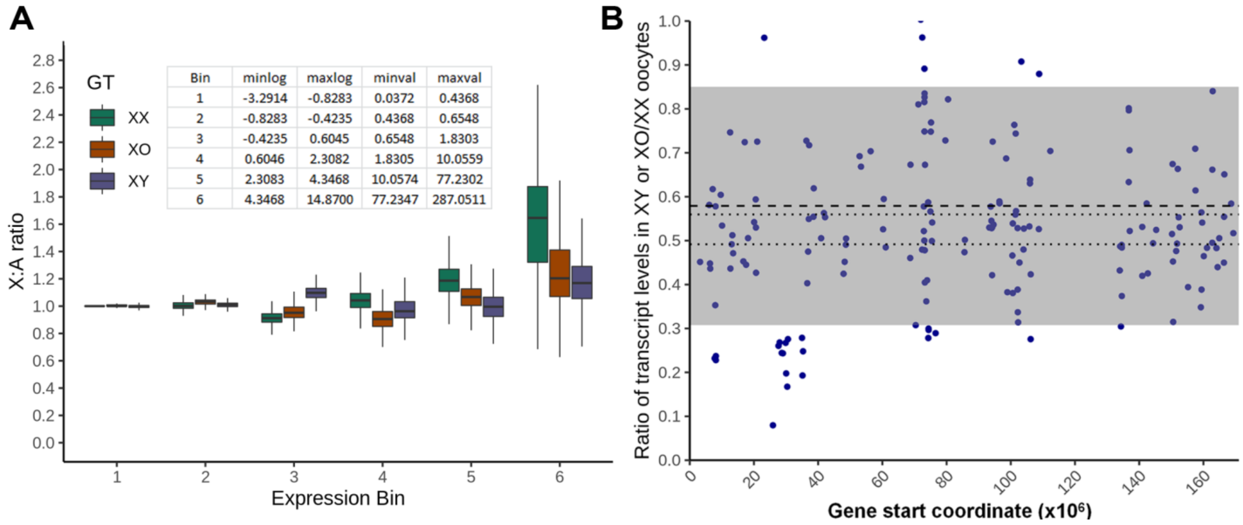

Bin	minlog	maxlog	minval	maxval
1	-3.2914	-0.8283	0.0372	0.4368
2	-0.8283	-0.4235	0.4368	0.6548
3	-0.4235	0.6045	0.6548	1.8303
4	0.6046	2.3082	1.8305	10.0559
5	2.3083	4.3468	10.0574	77.2302
6	4.3468	14.8700	77.2347	287.0511

Figure S2. X-linked gene dosage compensation against autosomal gene transcript levels. **A** The ratio of X-linked vs autosomal gene transcript levels, evenly divided into 6 Bins according to their transcript levels. Each box plot indicates the median with 1st & 3rd quartiles. The thin vertical line indicates the range from minimum to maximum values. **B** Ratio of the average transcript levels of all highly expressed X-linked genes in XO and XY oocytes against those in XX oocytes (Bin6 in **S2A**). The X-axis indicates the distance from the proximal end of the X chromosome. The thick broken line indicates the median, thin broken lines indicate the confidence of median and the gray area indicates the median ± SD.

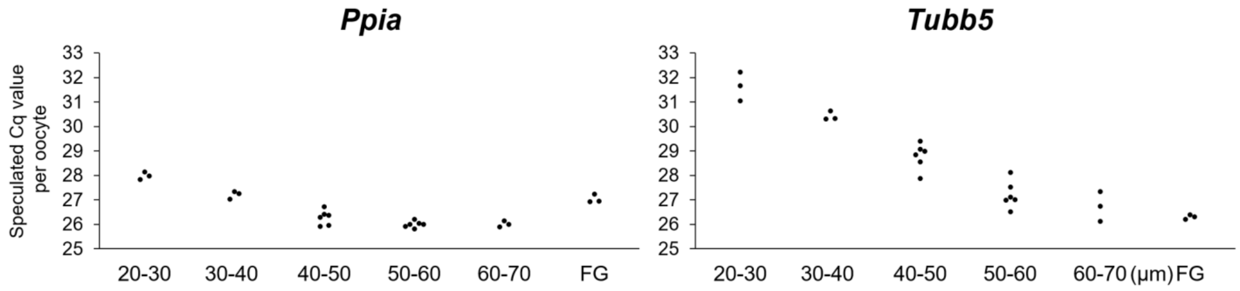

Figure S3. Changes in transcript levels of *Ppia* and *Tubb5* in XY oocytes during follicular growth. qRT-PCR Cq values detected in cDNA aliquots were adjusted to one XY oocyte of each diameter.

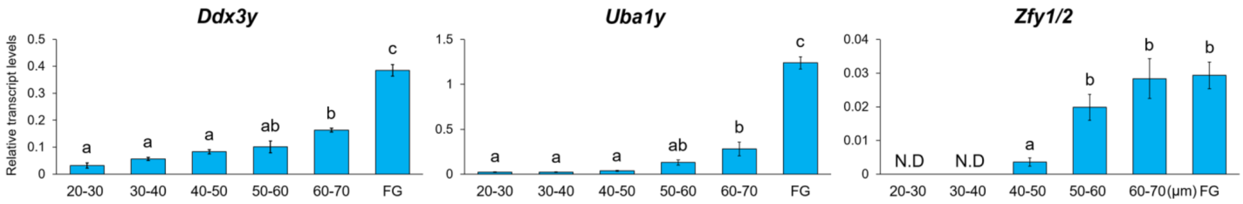

Figure S4. Changes in transcript levels of Y-linked genes in XY oocytes during follicular growth. Normalized to *Ppia* transcript levels. Different low case letters indicate significance at $P < 0.05$ by one-way ANOVA followed by Tukey's honestly significant difference (HSD) test.

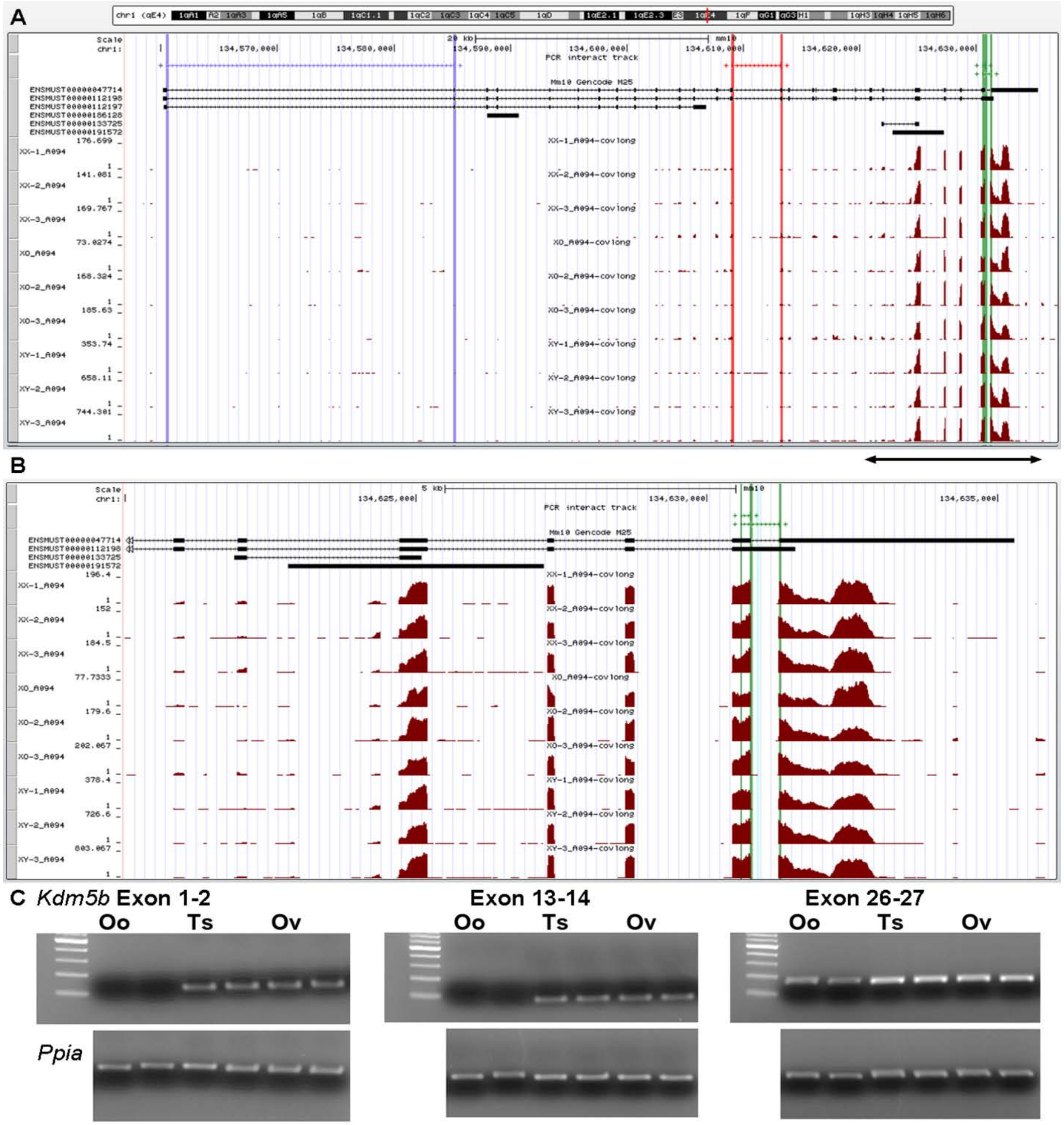

Figure S5. *Kdm5b* transcript analyses in oocytes in the growth phase. **A** RNA-Seq reads in the entire coding region. **B** Enlargement of the Exons 23-27 region indicated by arrows at the right bottom in **S5A**. **C** RT-PCR of *Kdm5b* and *Ppia* (control) in fully grown-oocytes (Oo), adult testes (Ts) and adult ovaries (Ov).
